# Supplementary material for: An Abl-FBP17 mechanosensing system couples local plasma membrane curvature and stress fiber remodeling during mechanoadaptation
Source: Nat Commun. 2019 Dec 20;10:5828. doi: 10.1038/s41467-019-13782-2 (PMC6925243; doi:10.1038/s41467-019-13782-2)
Supplement: Supplementary file 1 — Supplementary Information [file 41467_2019_13782_MOESM1_ESM.pdf]

An Abl-FBP17 mechanosensing system couples local plasma membrane curvature and stress fiber remodeling during mechanoadaptation

Echarri et al.

Supplementary Information

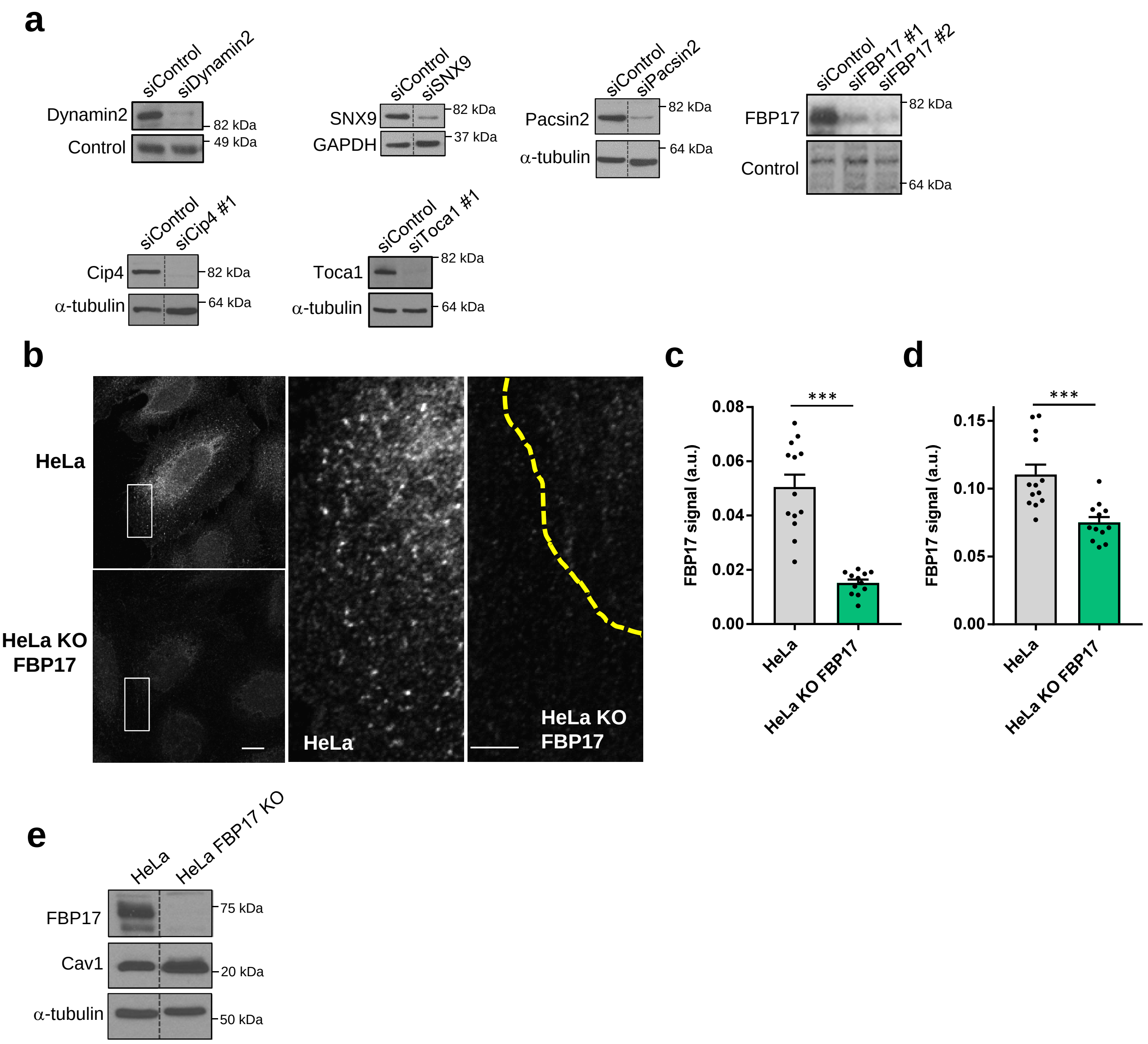

Supplementary Figure 1. Analysis of protein expression levels and antibody specificity.
a) Immunoblot showing specific suppression of various proteins in HeLa Cav1-GFP cells transfected with the corresponding siRNAs.
b) HeLa cells and gene-edited, FBP17 KO HeLa cells were stained for FBP17 (kindly provided by P. De Camilli). Non-specific weak signal was observed in the nuclear and perinuclear region area (delimited by a dotted yellow line). Scale bar 10 μm, box 5 μm.
c) Quantification of the staining in cytoplasmic region excluding the perinuclear area (see methods for details). N=13 (HeLa) and n=12 (HeLa KO FBP17) biologically independent cells, representative of 3 independent experiments. Statistical analysis with a two-tailed unpaired t test. ***P < 0.005.
d) Quantification of the staining in the nuclear region in HeLa and FBP17 KO HeLa cells. N=13 (HeLa) and n=12 (HeLa KO FBP17) biologically independent cells, representative of 3 independent experiments. Statistical analysis with a two-tailed unpaired t test. ***P < 0.005.
e) Immunoblot showing FBP17 levels in FBP17 KO Hela and control cells. Data represent mean ± S.E.M.

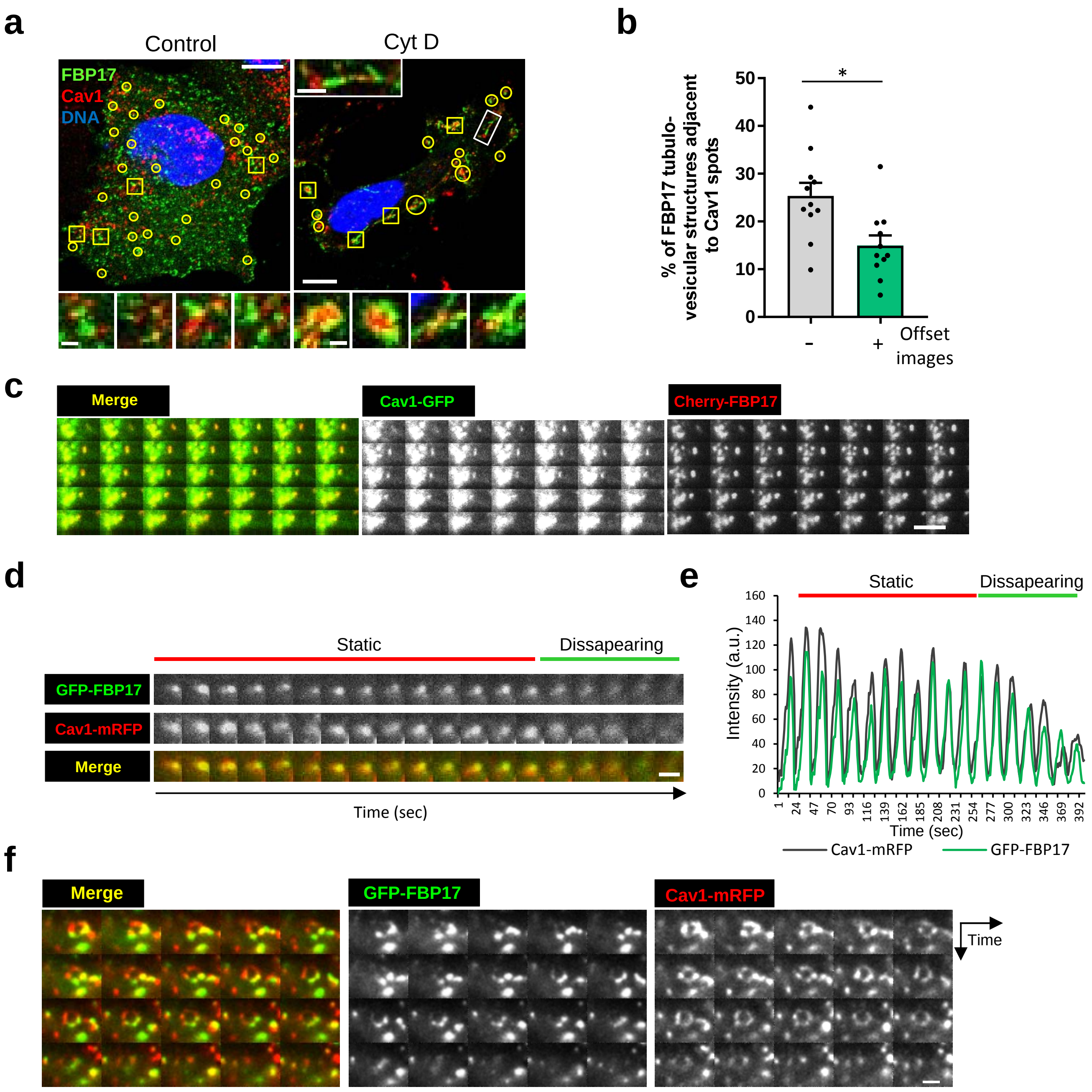

Supplementary Figure 2. Localization of GFP-FBP17 under different conditions.

a) Immunofluorescence of endogenous Cav1 and GFP-FBP17. Both proteins are frequently in an overlapping pattern or adjacent to each other (marked in yellow regions). Cytochalasin D (Cyt D, 1.25 μM for 60 min) and DMSO treated cells were fixed and examined by confocal microscopy. Both molecules cluster together (marked in yellow circles and squares corresponding to the insets) and intercalate in tubular structures (upper inset) in Cyt D treated cells. Scale bar 10 μm, low square images 1 μm, inset 2 μm.

b) Quantification of the overlapping/adjacent pattern between Cav1 and GFP-FBP17. The % of FBP17 positive spots or tubulo-vesicular structures overlapping with or adjacent to Cav1 spots was quantified in unmodified images and in images that were offset one respect to the other 8 pixels so that randomness of the overlapping pattern could be determined. N=11 biologically independent cells, representative of 3 independent experiments. Statistical analysis with a two-tailed unpaired t test. *P < 0.05. Data represent mean ± S.E.M.

c) Live imaging of Cav1-GFP and Cherry-FBP17 observed by TIRF. A vesicle positive for both molecules moves into a large cluster. Each image is 12.75 seconds apart. Scale bar 4 μm

d-e) Live cell imaging of GFP-FBP17 and Cav1-mRFP. Both molecules followed similar dynamics and disappear simultaneously from the TIRF plane. Quantification of the intensity of each vesicle in left panel is shown on the right. A line was drawn in the center of the image and along the time axis. Both molecules showed a similar dynamic behavior. Scale bar 1 μm.

f) Live TIRF imaging of clusters containing GFP-FBP17 and Cav1-mRFP. The clusters moved laterally as a whole unit. Images are 36 seconds apart. Scale bar 2 μm

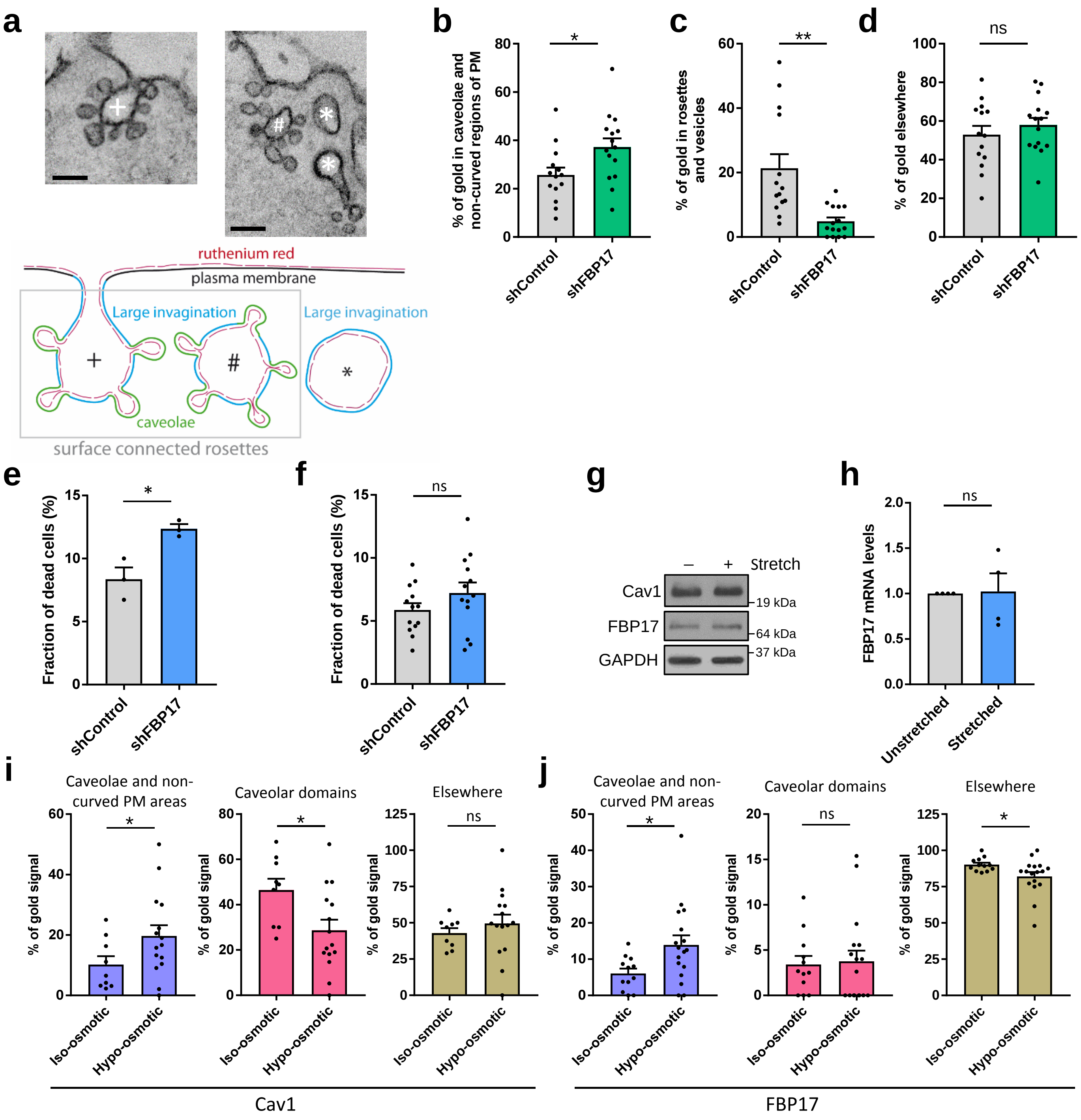

Supplementary Figure 3. Immuno-gold EM and sensitivity to mechanical stimuli experiments.

a) EM images and a diagram showing the different curved membrane invaginations analyzed. The upper left image shows a large invagination of the PM containing multiple caveolae, in this invagination the neck is clearly visible, the whole structure is a caveolar rosette (marked with a +), also shown in the drawing. In the right image a large invagination with multiple caveolae is observed but the neck was not visible. This structure also is a caveolar rosette and is surface-connected since it is labeled with ruthenium red (marked with an #). Surface-connected large invaginations without caveolae are also shown in the right image (marked with an *). In the bottom diagram the same structures are depicted. Scale bar 200 and 250 nm.

b-d) Quantification of immunogold signal observed by EM obtained using an antibody against endogenous Cav1. FBP17 silenced and control human fibroblasts were stained. N=14 (shControl) and n=15 (shFBP17) biologically independent cells, representative of 1 experiment. Statistical analysis with a two-tailed unpaired t test. *P < 0.05; **P < 0.01.

e) FBP17 expression was silenced in human fibroblast and cells were treated with hypo-osmotic medium for 2 minutes. Trypan blue labeled cells were scored and the fraction of dead cells positive for trypan blue were calculated in each condition. N=3 biologically independent samples representative of 3 independent experiments. Statistical analysis with a two-tailed unpaired t test. *P < 0.05.

f) FBP17 expression was silenced in human fibroblast and cells were stretched for 30 minutes. Scored as in e. N=13 biologically independent samples from 13 independent experiments. Statistical analysis with a two-tailed unpaired t test.

g) Immunoblot showing Cav1. FBP17 and GAPDH protein levels before and after 24 hours of stretching.

h) FPP17 mRNA levels (normalized to actin mRNA levels) before and after 48 hours of stretching. N=3 biologically independent samples from 3 independent experiments. Statistical analysis with a two-tailed unpaired t test.

i-j) Quantification of immunogold signal observed by EM obtained using an antibody against endogenous Cav1 (left) or FBP17 (right). The different regions of the cell analyzed are indicated. N=12 (iso) and n=17 (hypo) biologically independent cells representative of 1 experiment. Statistical analysis with a two-tailed unpaired t test. *P < 0.05. Data represent mean ± S.E.M. ns: non-significant.

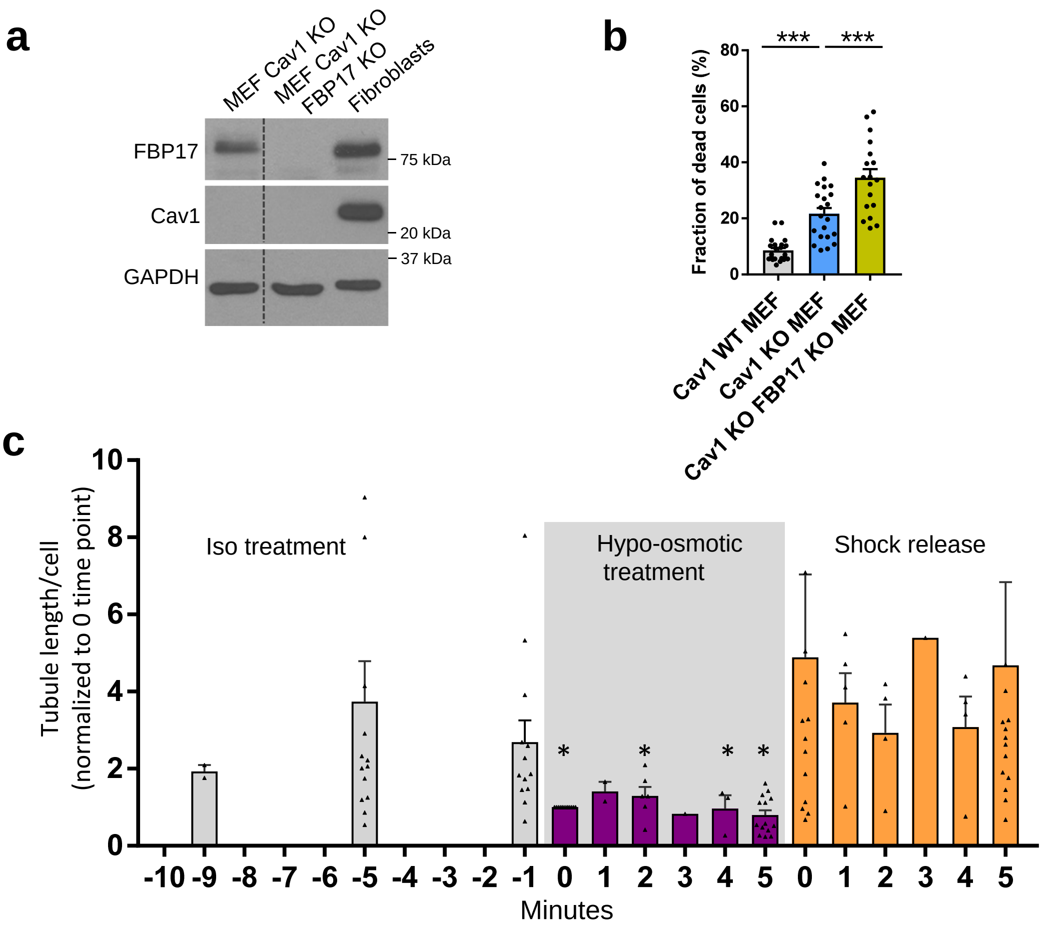

Supplementary Figure 4. Sensitivity of double FBP17 Cav1 KO cells and GFP-FBP17 to osmotic swelling.

a) Immunoblot showing specific suppression of FBP17 in Cav1 KO MEFs. Cav1 KO MEFs, human fibroblast and GAPDH are shown as controls.

b) The indicated cells were treated with hypo-osmotic medium for 10 minutes. Trypan blue labeled cells were scored and the fraction of dead cells positive for trypan blue were calculated in each condition. N=22 (MEF), n=21 (MEF Cav1 KO) and n=9 (MEF Cav1 KO FBP17 KO) biologically independent samples from the corresponding independent experiments. Statistical analysis with a two-tailed unpaired t test. ***P < 0.005.

c) FBP17 tubulation activity is sensitive to osmotic swelling**.** HeLa cells were transfected with GFP-FBP17 and recorded for 10 minutes before the media was diluted 1:10 with distilled water (30 mOsm, 5 minutes) and returned to iso-osmotic conditions for 5 minutes. The cells were imaged and the amount of GFP-FBP17-labeled tubules was scored. Statistical significance is indicated with respect to time -1 minute. Statistical analysis with a two-tailed unpaired t test. *P < 0.05. N information in sequential order starting from -10 min: 2, 14, 13, 14, 2, 6, 1, 3, 14, 13, 5, 4, 1, 4, 14. To visualize the data, three individual data points were excluded from the graph, these were 14.0 (-5 min), 29.8 (0 min shock release) and 32.5 (5 min shock release). Data represent mean ± S.E.M.

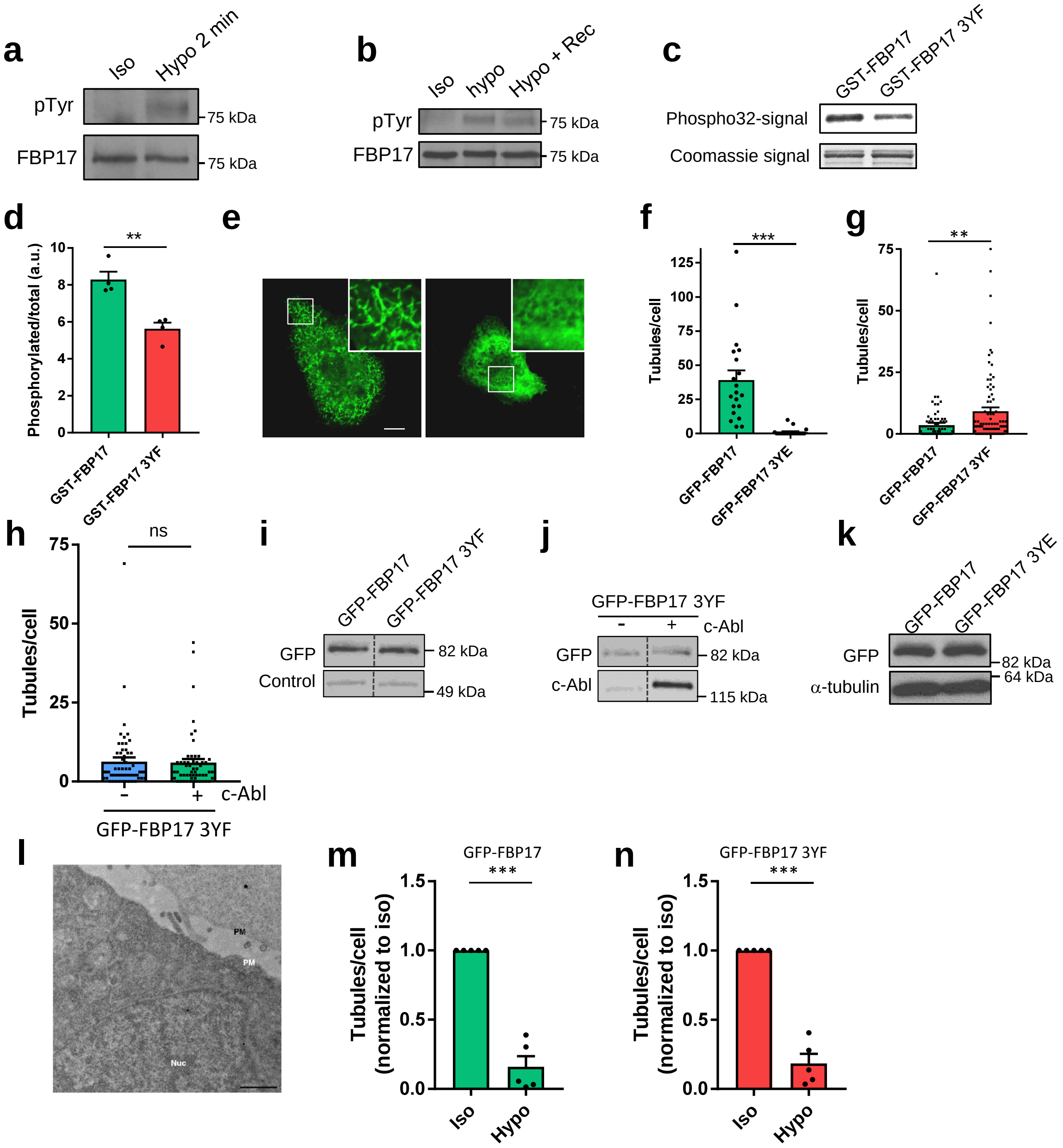

Supplementary Figure 5. Effect of tyrosine phosphorylation on FBP17 biological activity.

a) Human fibroblasts were treated with hypo-osmotic medium for 2 minutes and endogenous FBP17 was immunoprecipitated. Phosphotyrosine (pTyr) and total FBP17 were identified by western blot.

b) Human fibroblasts were treated with hypo-osmotic for 5 minutes or 5 minutes + 5 minutes in iso-osmotic media (Rec) and endogenous FBP17 was immunoprecipitated. Phosphotyrosine (pTyr) and total FBP17 were identified by western blot.

c-d) Phosphorylation of GST-FBP17 and GST-FBP17 3YF by pure c-Abl in vitro. The phosphorylated and the coomassie stained signals were quantified (panel d). N=4 biologically independent samples from 4 independent experiments. Statistical analysis with a two-tailed unpaired t test. $^{**}P < 0.01$.

e-f) GFP-FBP17 and GFP-FBP17 3YE were expressed in FBP17 KO HeLa cells and the amount of tubules calculated. N=20 biologically independent cells from 3 independent experiments. Statistical analysis with a two-tailed unpaired t test. $^{***}P < 0.005$. Scale bar 10 μm.

g) GFP-FBP17 and GFP-FBP17 3YF were expressed in HeLa cells and the amount of tubules/puncta per cell was calculated. Cells expressing low levels of protein were only analyzed. N=78 (GFP-FBP17) and n=86 (GFP-FBP17 3YF) biologically independent cells, representative of 3 independent experiments. Statistical analysis with a two-tailed unpaired t test. **P < 0.01.
h) GFP-FBP17 3YF was expressed with or without c-Abl and the amount of tubules per cell were quantified. N=57 (-) and n=55 (+) biologically independent cells representative of 3 independent experiments. Statistical analysis with a two-tailed unpaired t test. Ns: non-significant.
i-k) Immunoblot showing equal levels of the different forms of FBP17 under different conditions.
l) Localization of GFP-FBP17 3YE by EM. A diffuse pattern with no enrichment on caveolae or at the PM is observed. PM, plasma membrane, Nuc, nucleus. * marks a non-transfected cell. Scale 1 μm.
m, n) GFP-FBP17 and GFP-FBP17 3YF expressing cells were exposed to osmotic swelling (30 mOsm) for 10 minutes. N=5 biologically independent cells from 5 independent experiments. Statistical analysis with a two-tailed unpaired t test. ***P < 0.005. Data represent mean ± S.E.M.

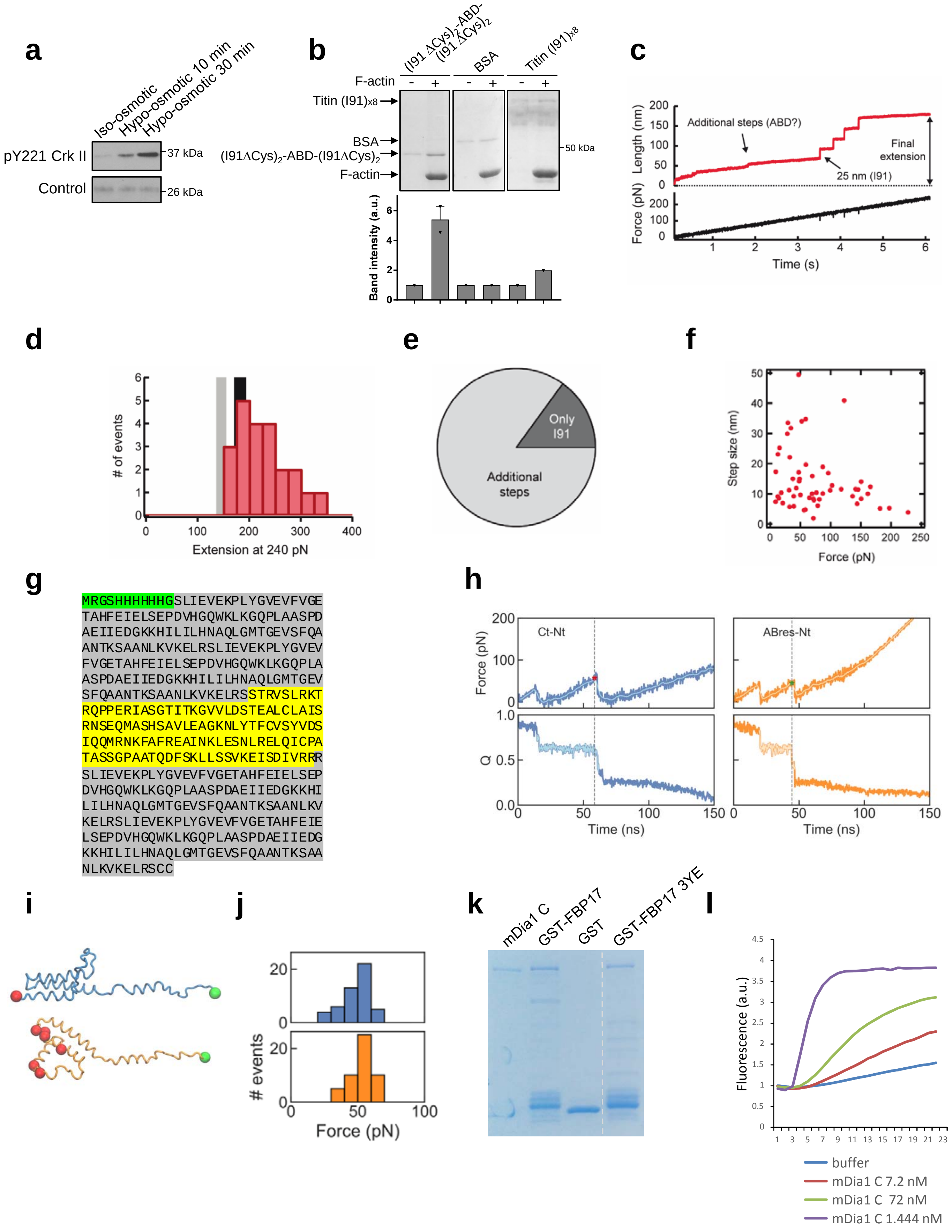
a
Iso-osmotic
Hypo-osmotic 10 min
Hypo-osmotic 30 min
pY221 Crk II
37 kDa
Control
26 kDa
b
(I91 ΔCys)2-ABD-(I91 ΔCys)2
BSA
Titin (I91)x8
F-actin
Titin (I91)x8
BSA
(I91ΔCys)2-ABD-(I91ΔCys)2
F-actin
50 kDa
Band intensity (a.u.)
c
Length (nm)
Additional steps (ABD?)
Final extension
25 nm (I91)
Force (pN)
Time (s)
d
# of events
Extension at 240 pN
e
Only I91
Additional steps
f
Step size (nm)
Force (pN)
g
MRGSHHHHHHGSLIEVEKPLYGVEVFVGE
TAHFEIELSEPDVHGQWKLKGQPLAASPD
AEIIEDGKKHILILHNAQLGMTGEVSFQA
ANTKSAANLKVKELRSLIEVEKPLYGVEV
FVGETAHFEIELSEPDVHGQWKLKGQPLA
ASPDAEIIEDGKKHILILHNAQLGMTGEV
SFQAANTKSAANLKVKELRSSTRVSLRKT
RQPPERIASGTITKGVVLDSTEALCLAIS
RNSEQMASHSAVLEAGKNLYTFCVSYVDS
IQQMRNKFAFREAINKLESNLRELQICPA
TASSGPAATQDFSKLLSSVKEISDIVRRR
SLIEVEKPLYGVEVFVGETAHFEIELSEP
DVHGQWKLKGQPLAASPDAEIIEDGKKHI
LILHNAQLGMTGEVSFQAANTKSAANLKV
KELRSLIEVEKPLYGVEVFVGETAHFEIE
LSEPDVHGQWKLKGQPLAASPDAEIIEDG
KKHILILHNAQLGMTGEVSFQAANTKSAA
NLKVKELRSCC
h
Force (pN)
Ct-Nt
ABres-Nt
Q
Time (ns)
i
j
events
Force (pN)
k
mDia1 C
GST-FBP17
GST
GST-FBP17 3YE
l
Fluorescence (a.u.)
buffer
mDia1 C 7.2 nM
mDia1 C 72 nM
mDia1 C 1.444 nM

Supplementary Figure 6. Characterization of c-Abl mechanosensitive properties and actin polymerization assay proteins.

a) c-Abl activation upon osmotic swelling (30 mOsm) in Src/Yes/Fyn KO MEFs. Endogenous CrkII was immunoprecipitated and pY221CrkII was detected. A loading control is shown.

b) In vitro F-actin binding properties of (I91 ΔCys)$_2$-ABD-(I91 ΔCys)$_2$. Pelleted proteins after centrifugation under the different conditions are shown. BSA and Titin I91x8 were used as negative controls. Quantification of the bands corresponding to (I91 ΔCys)$_2$-ABD-(I91 ΔCys)$_{2,}$ ABD, BSA and Titin I91x8 is shown below. N=2 for (I91 ΔCys)$_2$-ABD-(I91 ΔCys)$_2$. Data is represented as the mean $\pm$ S.E.M.

c) Typical force-ramp trace, in which 4 unfolding steps of 25 nm length mark the unfolding of the I91 protein modules.

d) The final extension at 240 pN for single molecule tethers was recorded from fingerprinted force-ramp traces containing four I91 unfolding events. Reference bars show the theoretical extensions if ABD unfolds during the experiment (black) or remains folded (gray), considering expected contour lengths and persistence lengths between 0.2 and 2.3 nm.

e) Proportion of traces that show additional steps in traces featuring four I91 unfolding events (n=19).

f) Scatter plot of steps that do not originate from I91 unfolding events showing their size and the force at which they occur.

g) (I91 ΔCys)$_2$-ABD-(I91 ΔCys)$_2$ polyprotein sequence. The tag (green), I91x2 (grey) and ABD (yellow) are shown.

h) Time series data from molecular simulations corresponding to the force (top) and the fraction of native contacts (Q, bottom) for two unfolding events. In the left panels (blue), the pulling force was applied to the protein ends, while in the right panels (orange) was applied to a selected group of residues putatively involved in actin binding and the N-terminus. In the bottom panels, the light color indicates the region of the transition path. In the top panels, stars mark the unfolding force.

i) Snapshots from the model corresponding to the point of the trajectory where the highest force was applied. Color code as in (h).

j) Histograms of unfolding forces from the simulations for the end to end pulling (blue) and alternative pulling (orange).

k) Coomassie staining of purified GST-FBP17 forms and mDia1 C.

l) In vitro actin-pyrene polymerization regulated by mDia1 C. Representative of three independent experiments.

Fig. 1b

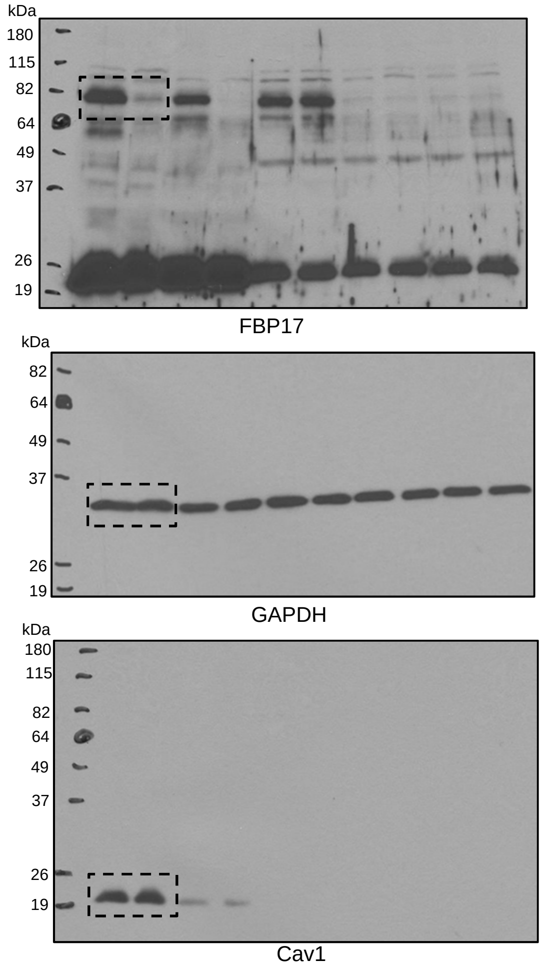

Fig. 3d

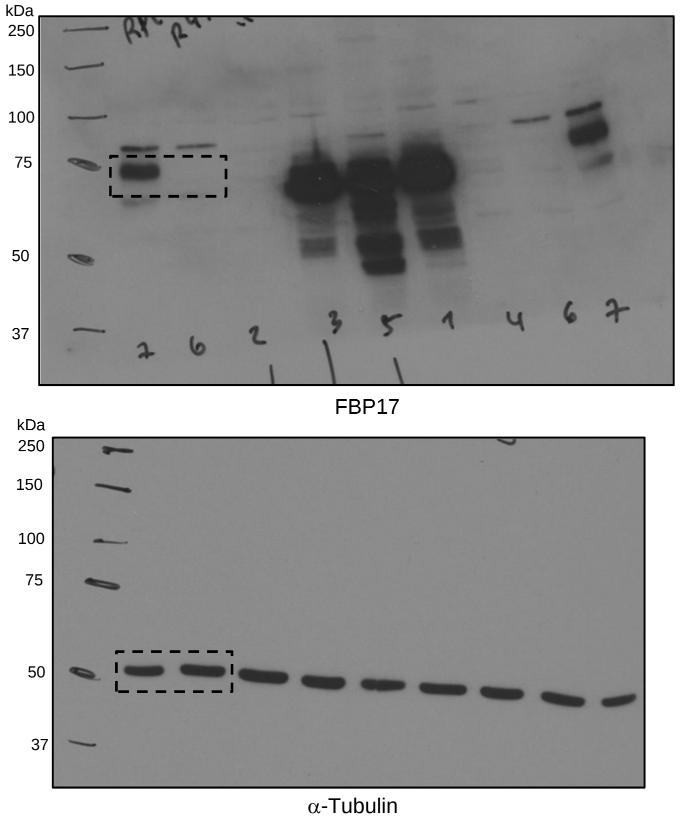

Fig. 5a

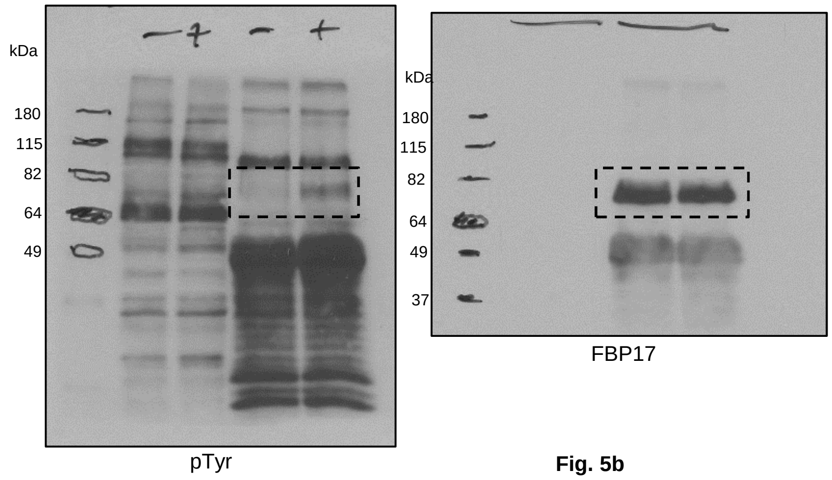

Fig. 5b

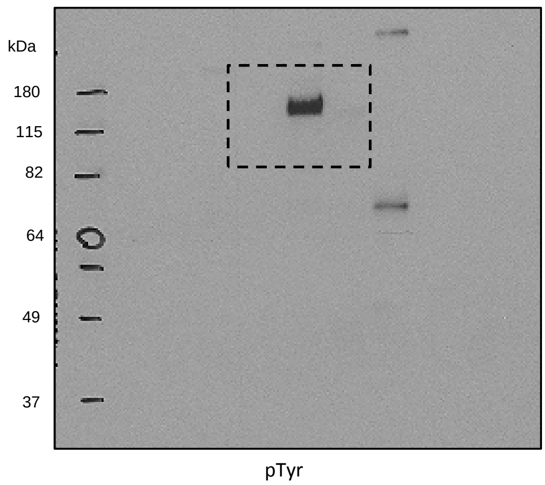

Fig. 5b

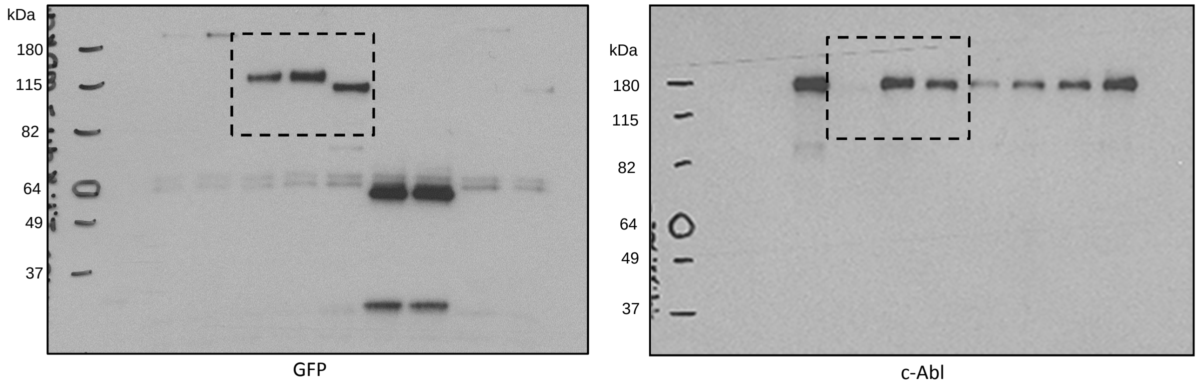

Fig. 5c

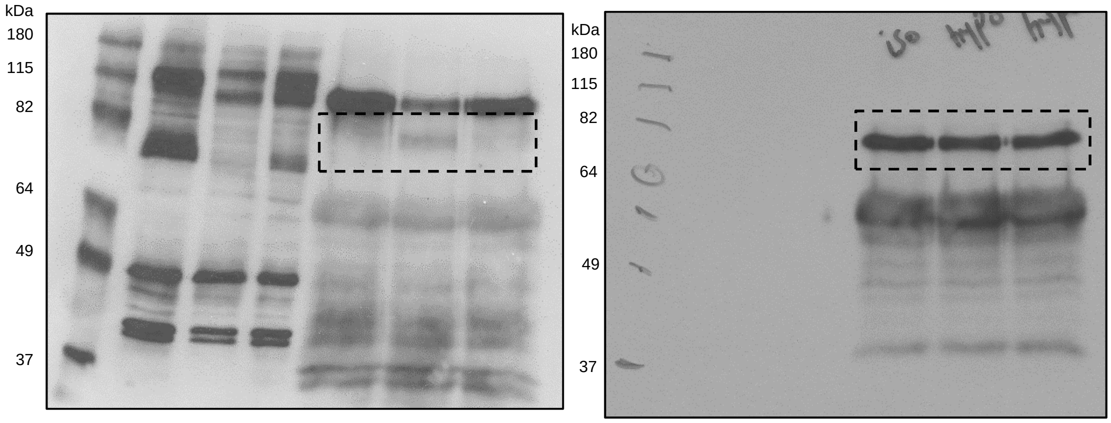

pTyr FBP17

Fig. 5e

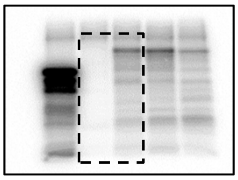

Phospho^{32} Signal

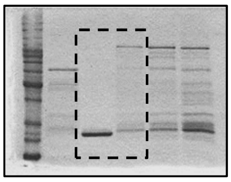

Coomassie

Fig. 5f

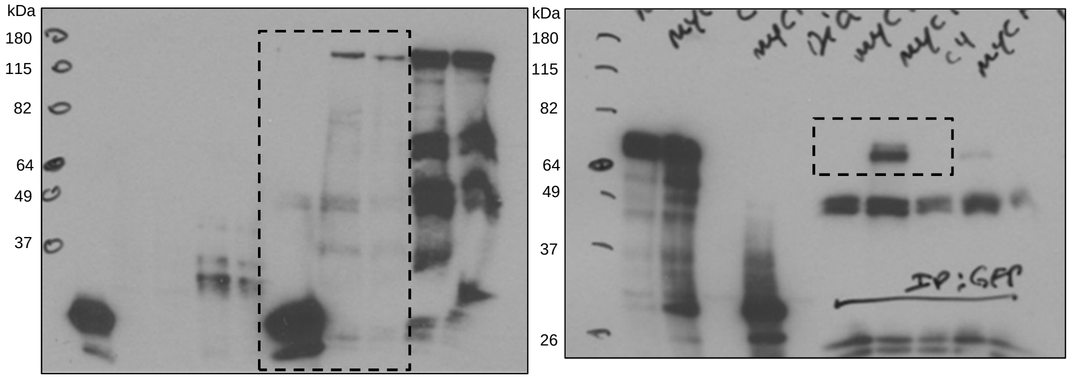

GFP Myc (FBP17)

Fig. 5f

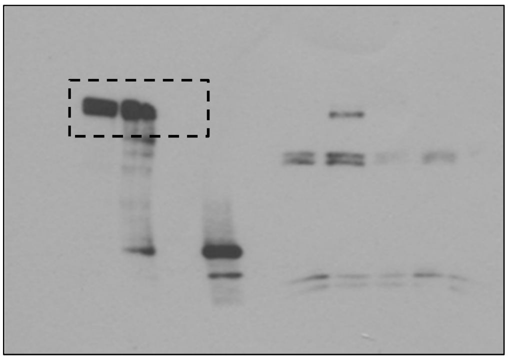

Myc (FBP17)

Fig. 5g

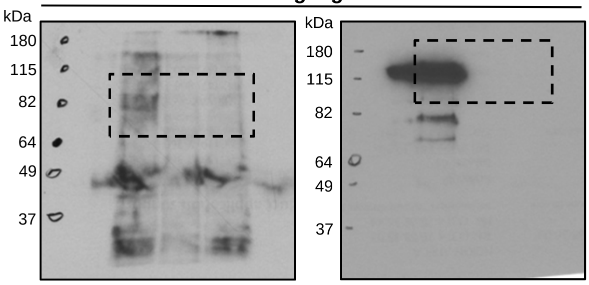

FBP17 c-Abl

Fig. 6c (order of bands inverted in fig. 6c cropped blots)

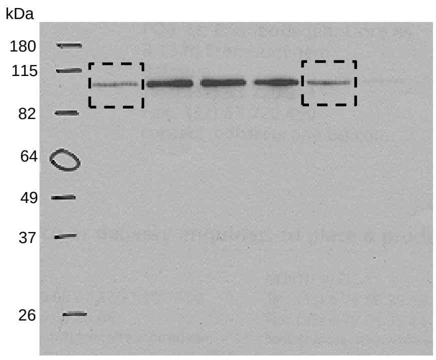

GFP

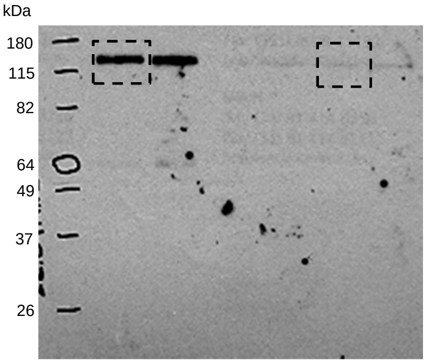

c-Abl

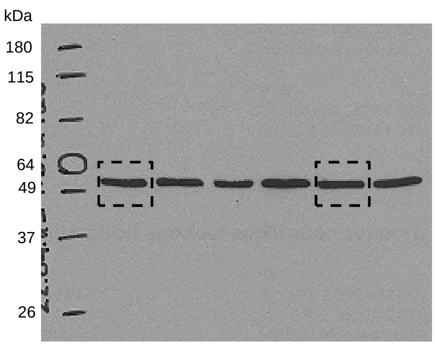

α-Tubulin

Fig. 6d

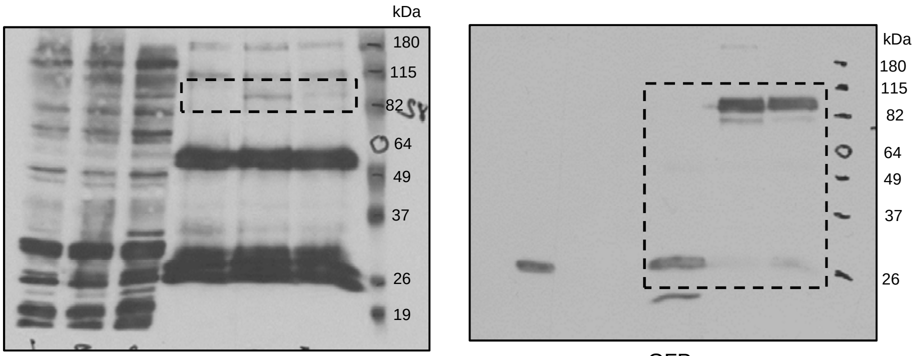

pTyr GFP

Fig. 6j

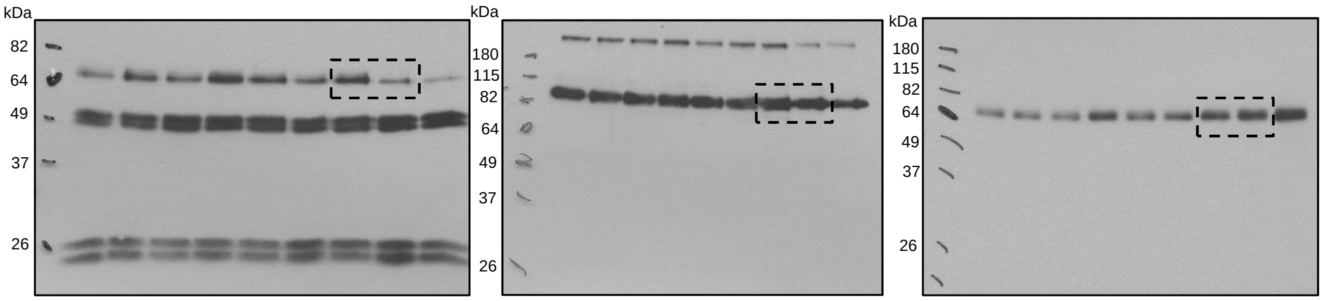

c-Myc GFP c-Myc

Fig. 7a

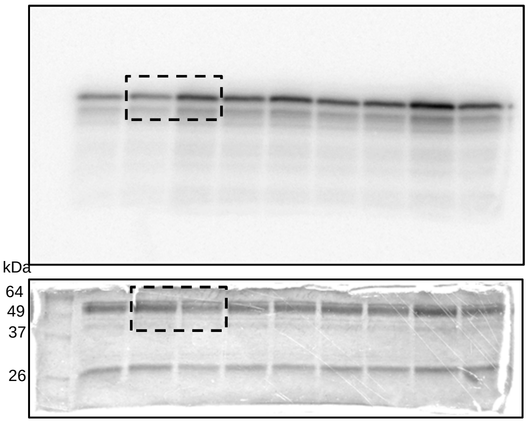

Fig. 7c

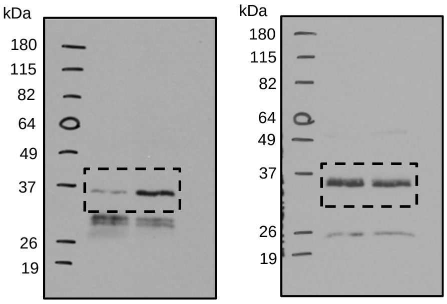

pY221CrkII Crk II

Fig. 7d

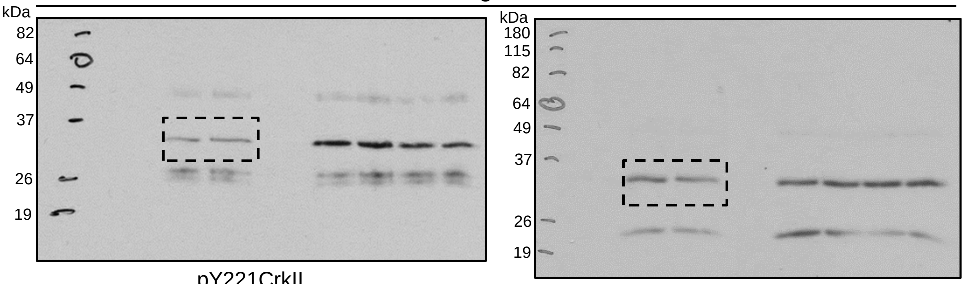

pY221CrkII Crk II

Supplementay Figure 1a

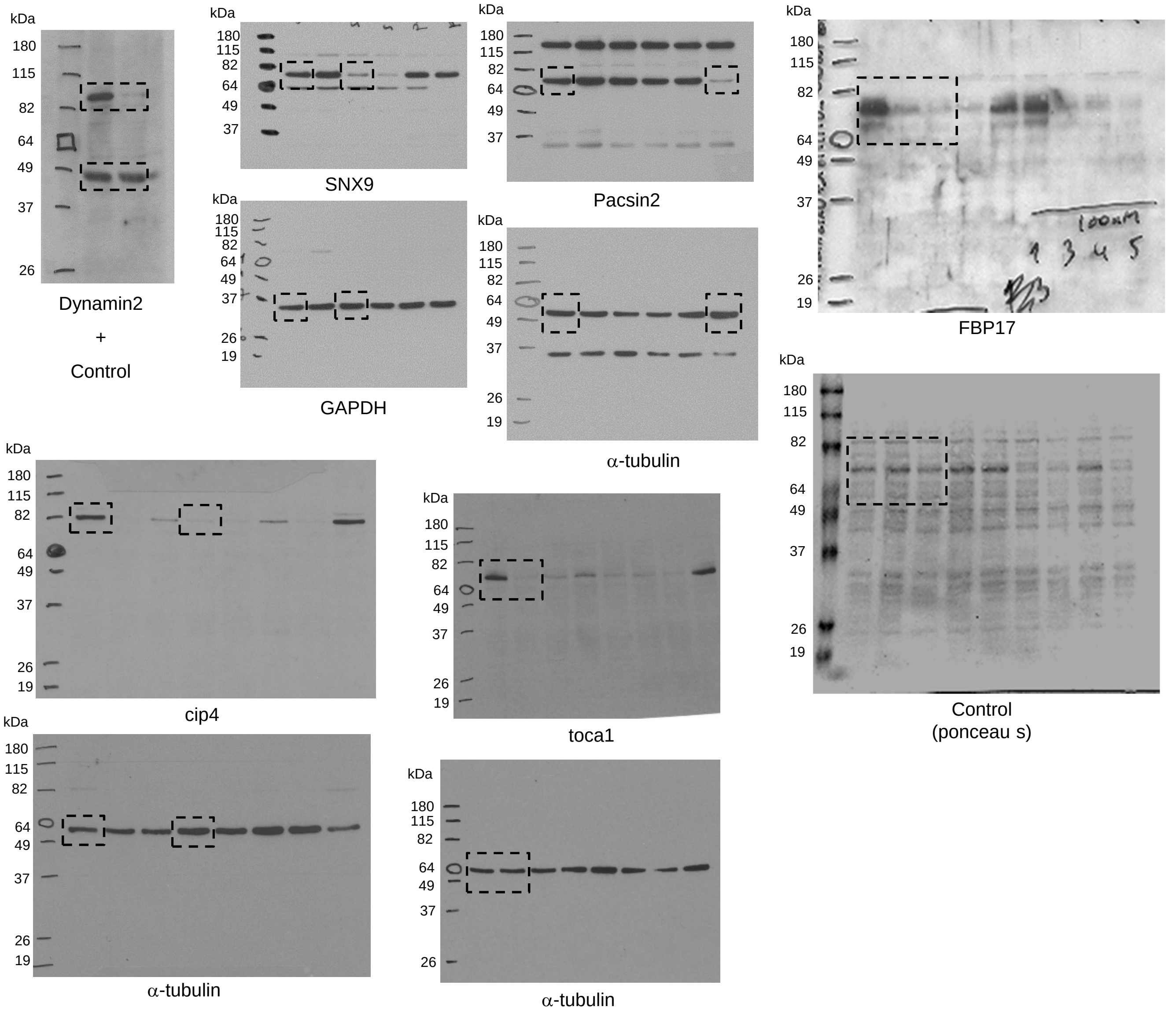

Supplementay Figure 1e

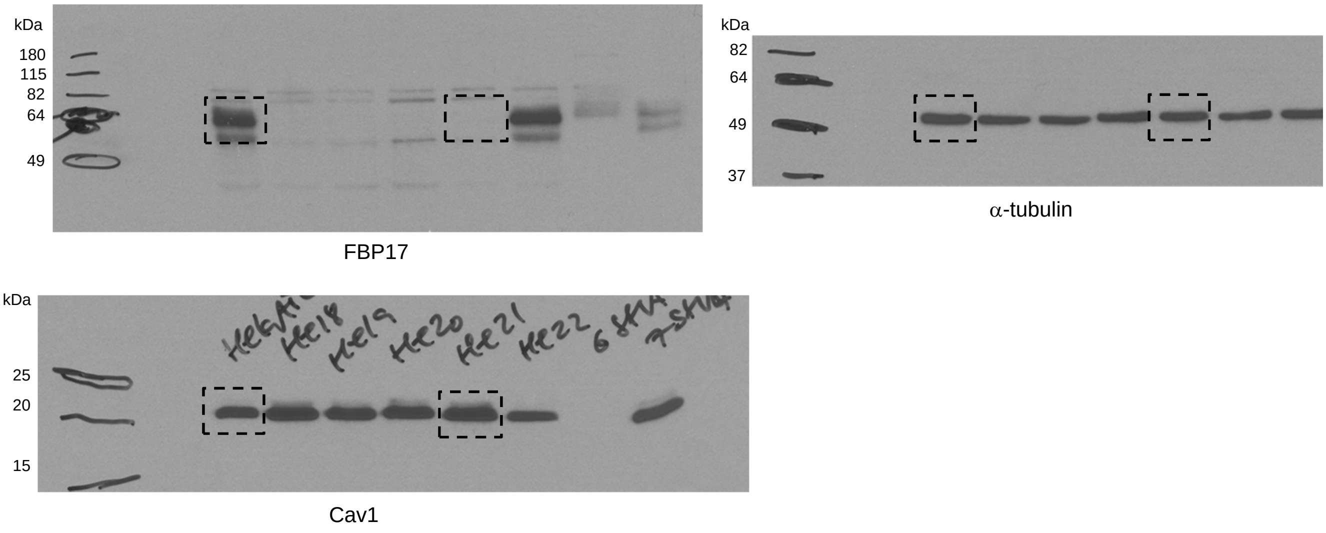

Supplementay Figure 3g

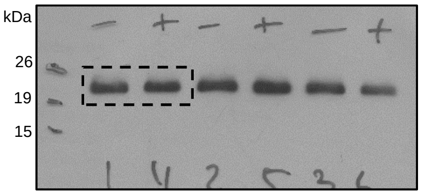

Cav1

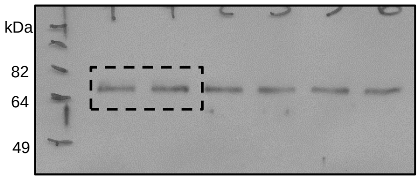

FBP17

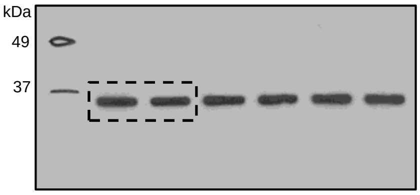

GAPDH

Supplementay Figure 4a

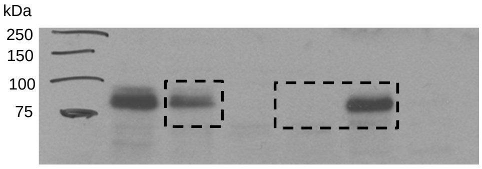

FBP17

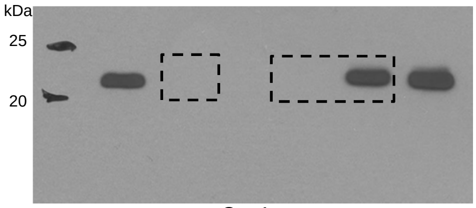

Cav1

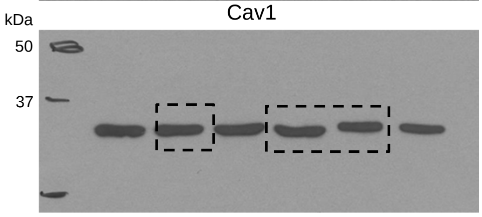

GAPDH

Supplementay Figure 5i (lane order inverted in cropped fig. 5i)

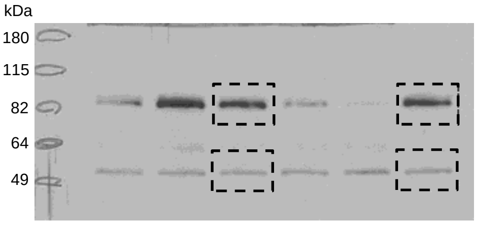

GFP

Supplementay Figure 5b

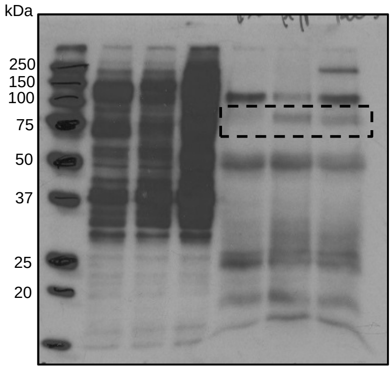

pTyr

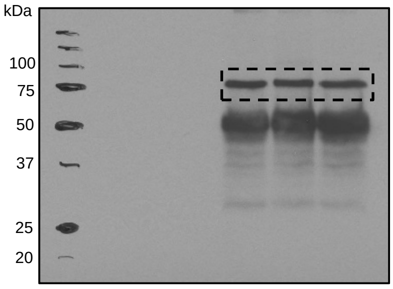

FBP17

Supplementay Figure 5k

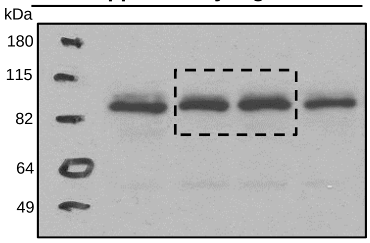

GFP

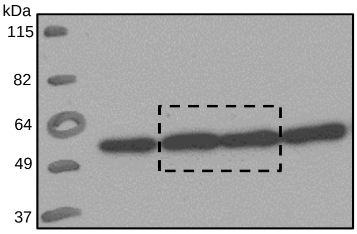

α-tubulin

Supplementay Figure 5a

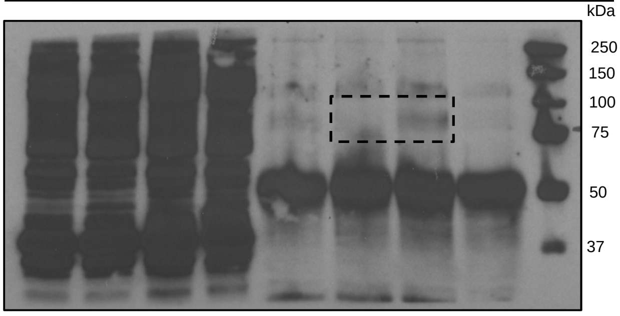

pTyr

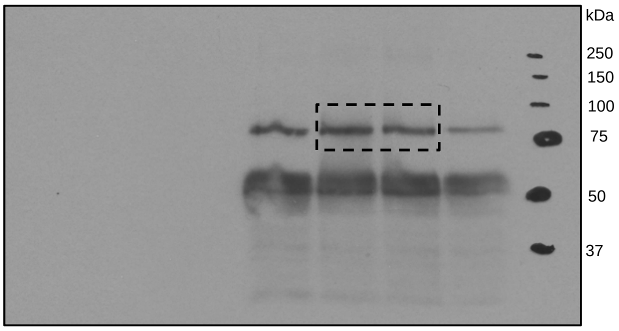

FBP17

Supplementay Figure 5c

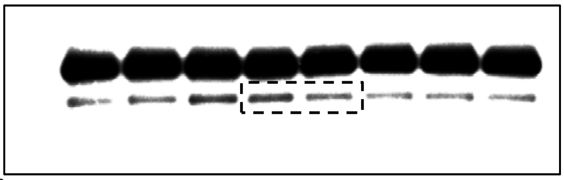

Phospho32-signal

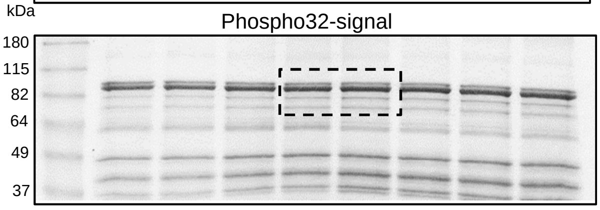

Coomassie signal

Supplementay Figure 5j (lane order inverted in cropped fig. 5j)

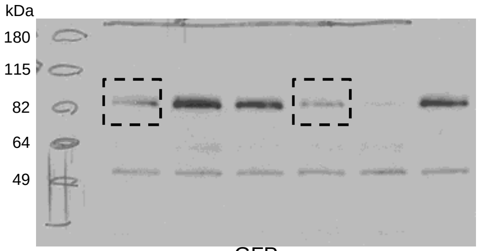

GFP

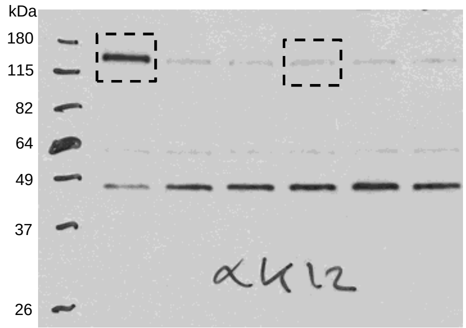

c-Abl

Supplementaty Figure 6a

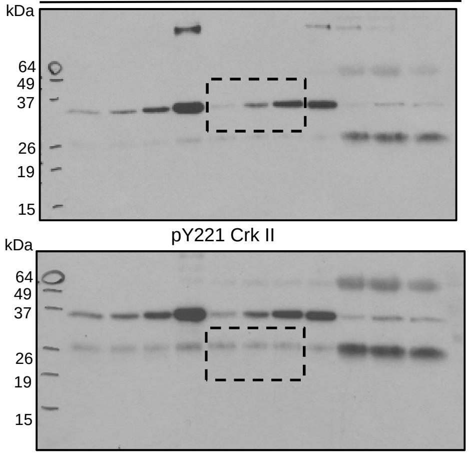

Control

Supplementaty Figure 6b

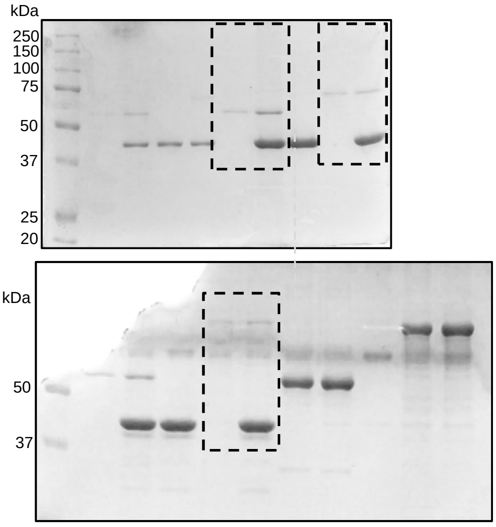

Coomassie gels

Supplementaty Figure 6k

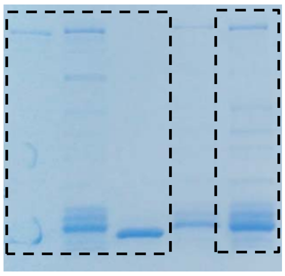

Coomassie gel

Supplementary Figure 7. Full scans of western blots, radioactivity generated images and coomassie-stained gels.

Supplementary Methods

Immunoprecipitation

To precipitate endogenous FBP17, cells were kept in iso-osmotic or hypo-osmotic conditions for 10 minutes. Cells were lysed in RIPA buffer (50 mM Tris-HCl pH 7.5, 150 mM NaCl, 1% Triton-x-100, 0.1% SDS, 1% sodium deoxycholate) in the presence of protease and phosphatase inhibitors; phosphatase inhibitor sodium orthovanadate (0.2 mM) was added during the treatment and previous to the lysis to prevent dephosphorylation. Lysates were spun and incubated with control or FBP17 790 antibody for 2 h and then complexes were collected by incubation with protein A-sepharose. To detect phospho-Y221-CrkII (pY221CrkII), a readout of c-Abl activity [1], CrkII was precipitated using RIPA as lysis buffer. Co-immunoprecipitations of endogenous c-Abl (with K-12 antibody) and FBP17 (Fig. 5g) and co-expressed tagged FBP17 (Fig. 5f) were done in KLB (20 mM Tris-HCl, pH 7.5, 150 mM NaCl, 1% Triton-x-100) + protease and phosphatase (pp) inhibitors.

In vitro kinase assay

Endogenous c-Abl was immunoprecipitated using K-12 antibody in IP buffer (50 mM Hepes pH 7.0, 150 mM NaCl, 10% glycerol, 1% Triton-x-100, 1.5 mM $MgCl_2$, 1 mM EGTA + inhibitors), extensively washed twice with RIPA + pp inhibitors, buffer 2 (10 mM Tris-HCl pH 7.4, 5 mM EDTA, 100 mM NaCl, 1% Triton-x-100 + pp inhibitors), buffer 3 (10 mM Tris-HCl pH 7.4, 5 mM EDTA, 1% Triton-x-100) and buffer 4 (20 mM Tris-HCl pH 7.4, 10 mM $MgCl_2$ and 1mM DTT). Immunopurified c-Abl was incubated in buffer 4 with GST-CrkII in the presence of [γ-^{32}P] ATP for 45 minutes. Samples were resolved on SDS-PAGE and radioactive signal was collected in a Phospho-Imager screen. Total GST-CrkII protein was simultaneously visualized by coomassie staining.

Polyacrylamide based hydrogels

Hydrogels were built with different polyacrylamide:bisacrylamide concentrations to reach a rigidity of 0.2 kPa (7.5% acrylamide and 0.01% bis-acrylamide) and 55 kPa (12% acrylamide and 0.6% bis-acrylamide). Briefly, Silene-coated and sigmacote-coated coverslips were used to sandwich the polyacrylamide:bisacrylamide mixtures. Sulpho-Sampah was used to activate the solidified hydrogels and they were coated with fibronectin (5 μg/ml) before cells were plated for 24 hours [2].

Adhesion-regulated Cav1 inward trafficking

Cells were trypsinized and transferred to methylcellulose solution (0.5% (w/v) methylcellulose, 0.2% (w/v) lipid free bovine serum albumin, and 0.5 mg/ml soybean trypsin

inhibitor). Cells were rotated at 37ºC for 1 hour (Fig. 1a) or 5 and 20 minutes (Fig. 1c). To quantify the cell edge pool of endogenous Cav1, the PM region was labeled with wheat germ agglutinin and the amount of Cav1 disappearing from this region from 5 minutes to 20 minutes of suspended cells was set at 100% endocytosis in control cells [3].

Generation of FBP17 KO cell lines

The FBP17 null stable cell lines were generated using plasmids based CRISPR/Cas9 genome editing, using two vectors: pX330-U6-Chimeric_BB-CBh-hSpCas9 (addgene # 42230) and pSpCas9(BB)-2A-GFP (PX458) (addgene # 48138). Four vectors, two targeting human and two targeting mouse *fbp17* were designed and generated. The design of the sgRNAs was carried out using the MIT database (http://crispr.mit.edu). In each case (mouse or human), we cloned two sgRNAs for the gene, one in each vector, and transfected both using Lipofectamine 3000 (Thermo Fisher). The transfected cells were sorted by GFP signal in one-cell per well p96 plates and the clones were analyzed by western blot against FBP17. Oligos for hFBP17: sgDNA1: Fw: CACCGTCCCCTGCACCATGAGCTG; Rv: AAACCAGCTCATGGTGCAGGGGAC. sgRNA2: Fw: CACC GCTCGGGCCAAGGCGGACGA; Rv: AAACTCGTCCGCCTTGGCCCGAGC. Oligos for mFBP17: sgDNA1: Fw: CACC CCGGTCCCTGCACCATGAGC; Rv: AAAC GCTCATGGTG CAGGGACCGG. sgDNA2: Fw: CACC ACGCCCGCGGGTTGCCGCTC; Rv: AAACGAGCGGCAACCCGCGGGCGT.

Statistical analysis

Mean values were compared by two-tailed unpaired Student's *t* test. P values below or equal to 0.05, 0.01, or 0.005 were considered statistically significant and were labeled with 1, 2 or 3 asterisks respectively. Data is represented as the mean ± s.e.m., unless otherwise indicated. Mann-Whitney non-parametric test was used to compare the complete distribution of vesicle intensities across conditions. To compare proportions, a proportions test based on a Chi2 test was used.

Microscopy and image analysis

Images were acquired on a Zeiss LSM 700, a Leica TCS SP5 (confocal) or a Leica AM TIRF MC (TIRF) with a 100x, 1.46 NA objective. Image J was used to adjust brightness and contrast values. For the adjacent localization analysis of FBP17 and Cav1, spots with ≥4 pixels were manually identified and adjacent Cav1 spots to FBP17 spots were defined as they were overlapping or a continuation of the signal. For the offset modification of the images to account for random signal overlap (Fig. 1e, Supplementary Figure 2b, 5i), the image of one channel was moved to the left 8 pixels and merged with the other channel image. Live imaging

of GFP-FBP17 tubules under hypo-osmotic shock were recorded in TIRF and EPI-fluorescence modes. Epi-fluorescence images, that also contained the TIRF plane, were quantified. Images with FBP17-induced tubules were blind-scored manually or by an automated analysis using Definiens software. Different types of stress fibers were counted manually blind-scored as defined [4, 5]. To identify Cav1 clusters (Fig. 3a) Cav1 spots were segmented in a defined region and the integrated intensity was quantified in each image. The intensity profiles were divided into 4 quartiles in the control sample and the % of spots in the treated cell falling into each quartile was calculated. FBP17 antibody specificity was determined using FBP17 KO cells. Wild type cells and KO cells were stained for FBP17 using the same conditions as in Itoh et al. [6], and the signal was quantified in the nuclear area, where it gave unspecific signal and outside this region where the signal was specific. The analysis of the colocalization between FBP17 and Cav1 excluded the nucleus and its immediate region, where the antibody gave unspecific signal. The co-localization between FBP17 and c-Abl was estimated by computing the Pearson´s coefficient.

Electron microscopy

HeLa cells were processed for EM following standard procedures. Briefly, cells were fixed in 0.1 M cacodylate buffer, pH 7.4 containing 2.5% glutaraldehyde (+1 mg/ml ruthenium red), and then post-fixed in 1% osmium tetroxide (+1 mg/ml ruthenium red), followed by treatment with 2% uranyl acetate. The samples were dehydrated, embedded in LX112 Epon resin, sectioned and stained. To quantify the number of caveolae per sample, the total membrane length analyzed was estimated using ImageJ software. Morphologically defined single caveolae at the PM or invaginations containing caveolae (herein rosettes) connected with the PM (ruthenium red positive) were scored. Similarly, large invaginations with no caveolae and ruthenium red positive were scored. GFP-tagged constructs were detected using APEX-GBP [7]. For Immuno-gold labeling, samples were fixed in 2% PFA, 0.2% glutaraldehyde, in 0.1 M phosphate buffer pH 7.4. The cells were embedded in 10% (wt/vol) gelatin and cryoprotected overnight in 2.3 M sucrose. Specimens were rapidly frozen in liquid nitrogen and cryosectioned with a Leica EM FCS cryoultramicrotome at −120°C. For immunogold labeling, thawed 70-80 nm-thick cryosections were incubated with 20 mM glycine for 5 min to quench the free aldehyde groups and with 10% FBS for 5 min to block nonspecific binding. Then, primary rabbit antibodies were incubated for 30 min at RT, followed by addition of protein A conjugated to 10-nm gold particles (EM Laboratory). Sections were stained with a mix of 1.8% methylcellulose and 0.4% uranyl acetate before their visualization. Specimens were examined at 80 kV with a JEOL JEM-1010 equipped with TVIPS F416 camera. Cav1 was stained with Cav1

XP and FBP17 rabbit serum [6].

Liposome formation

Large liposomes were formed by mixing 16:0 liss rhodamine PE (810158, Avanti Polar lipids, Inc. at 1 mg/ml in chloroform) and bovine brain extract (SIGMA-Aldrich B1502, at 1 mg/ml in chloroform) so that the proportion is 5% and 95% respectively in 100 ul final volume. The mixture was dried under nitrogen for 30 minutes and under vacuum for 2h. It was resuspended in 100 ul (final concentration 1 mg/ml) of 0.3M sucrose and incubated 1h at 37C. Vortexed for 2 minutes. Liposomes were diluted in 1/40 in 20 mM Hepes pH 7.5, 100 mM KCl (buffer L1) and 1 mM DTT. At this stage liposomes were 1-10 μm in diameter.

In vitro tubulation assay

Purified proteins dialyzed against buffer L1 were incubated at 0.15 μg/μl (1.2 μM final concentration) with 0.05 μg of liposome mixture in final volume of 40-50 μl. Mixtures were incubated at room temperature for 5 minutes and aliquots spotted on glass slides were observed under the epifluorescence microscope immediately. The amount of liposomes per field was estimated based on the quantification of 3 random fields in each experiment and condition. The amount of tubules in 20-50 fields were counted and the fraction of tubulated liposomes was estimated in each condition.

F-Actin binding/polymerization assays

For F-actin polymerization assay, rabbit skeletal muscle (cytoskeleton, inc.) was polymerized as indicated by the manufacturer. F-actin at 9 μM was incubated with the protein of interest at 25 μM (in 20 mM HEPES, pH 7.2, 150 mM NaCl, 1 mM EDTA) in buffer A: 20 mM imidazole, pH 7.0, 150 mM NaCl, 2 MgCl2, 0.5 mM ATP, 1 mM EGTA, 1 mM DTT) in a final volume of 60 μl. This reaction was incubated for 30 minutes at room temperature and spun at 100.000 g for 20 minutes. Pellets were run in a SDS-PAGE gel and stained with coomassie. For F-actin polymerization assay, mDia1 C-terminus (mDia1 C: amino acids 549-1255) was purified [8]. The protein mixtures containing mDia1 C and GST-FBP17, GST-FBP17 3YE or GST were pre-incubated for 30 minutes at 4°C and incubated with pyrene-G-actin as indicated by the manufacturer (cytoskeleton, inc.). All proteins were dialyzed in 150mM NaCl, 0.1 mM MgCl, 0.1 mM EGTA, 2 mM phosphate buffer pH 7 and 1 mM DTT [8]. F-actin polymerization was monitored by the fluorescence emitted by pyrene-actin at 405 nm when incorporated into filamentous actin.

Force measurements

PM tethers were extracted from cells by a concanavalin A (Sigma-Aldrich) coated bead (3 µm in diameter, Polysciences) trapped in optical tweezers. The optical tweezers are made of a 1064 nm laser beam (ytterbium fiber laser, λ = 1064 nm, TEM 00, 5 W, IPG Photonics, Oxford, MA) expanded and steered (optics by Elliot Scientific, Harpenden, UK) in the back focal plane of the microscope objective (Apo-TIRF 100× NA 1.45, Nikon). The whole setup was mounted on a Nikon Eclipse-Ti inverted microscope. The sample was illuminated by transmitted light, and movies were acquired at 10 Hz with an EM-charge-coupled device camera (Andor iXon 897) driven by Micro-Manager [9]. The fine movements and particularly the translational movement necessary to pull the membrane tether were performed using a custom-made stage mounted on a piezoelectric element (P753, Physik Instrumente, Karlsruhe, Germany) driven by a servo controller (E665, Physik Instrumente) and a function generator (Sony Tektronix AFG320). Calibration was performed using an oscillatory modulation driven by a function generator [10] and measuring the response of the bead to an oscillatory motion of the stage. Their relationship is linear in the laser power range used for the experiments (0.5–2 W).

The membrane tether was held at constant length to measure the static force. For measuring membrane tension changes due to hypo-osmotic shock, a second tether was pulled 5 minutes after the medium was diluted diluting growth medium with deionized water (to 60 mOsm). The position of the beads used to compute tether forces was detected from the images using a custom ImageJ macro.

Single-molecule force-spectroscopy by atomic force microscopy

AFM experiments were done in a Luigs and Neumann AFS following standard procedures [11]. Briefly, 1-10 uL of purified (I91 ΔCys)$_2$-ABD-(I91 ΔCys)$_2$ protein were deposited on a gold cover slip. To ensure attachment to the gold cover slip, the (I91 ΔCys)$_2$-ABD-(I91 ΔCys)$_2$ contains two terminal cysteine residues that form thio-gold covalent bonds with the surface. To pick up single molecules, the cantilever tip (MLCT-C, Bruker; spring constants were obtained by the thermal fluctuations methods [12] and were 15-20 pN/nm) was pushed against the surface at forces between 800 and 2000 pN, for up to 2 seconds, and then the piezo motor was retracted at a constant speed of 400 nm/s (force extension), or to reach the predefined force set point (40 pN/s force ramp). Successful single-molecule events are fingerprinted by the presence of 4 unfolding events corresponding to I91 ΔCys domains and detachment forces of at least 240 pN. Contour lengths in force-extension experiments were estimated using the worm-like chain model of polymer elasticity [13]. Theoretical final contour lengths were calculated considering the extension of unfolded I91 domains (89 amino acids), 19 linker and extra amino acids in (I91 ΔCys)$_2$-ABD-(I91 ΔCys)$_2$, and whether the ABD domain remains folded

or not. To estimate the contour length of folded ABD, we considered the 2.3 nm distance between alpha carbons of residues Ser31 and Arg137 in the solution structure of human ABD, and that the first 17 ABD amino acids in (I91 ΔCys)$_2$-ABD-(I91 ΔCys)$_2$ are unfolded (Supplementary Figure 6g). We considered that the contour length per unfolded amino acid is 0.4 nm [14]. To estimate final extensions at 240 pN, we used the worm-like chain model of polymer elasticity using extreme values of persistence length between 0.2 and 2.3 nm [15]. Analysis was done using custom-written software in Igor Pro 6 (Wavemetrics).

Coarse grained molecular dynamics simulations

We have performed molecular simulations of the unfolding of ABD upon force using the coarse-grained model by Karanicolas and Brooks [16], which has been extensively used in the context of unfolding upon force [17]. Briefly, this is an explicit chain model where amino acid residues are represented as beads whose centers are located in the alpha-carbon of the protein and interactions between residue pairs are dictated by the contacts in the experimental structure (i.e. whether a pair of residues have heavy atom pairs closer than 4.5 Å). All pairs of beads not in contact exclude each other through a generic repulsive interaction potential. Harmonic terms are defined in the energy function for bonds and angles, based on the reference structure, and a statistical potential is used for torsions. In this case, we generated parameters using the most representative model from the NMR structure of the F-actin binding domain of human Brc-Abl/c-Abl (1zzp).

Molecular simulations were performed at 300 K using the Gromacs 4.0.5 package [18] using a stochastic dynamics integrator with a time-step of 10 fs and friction constant of 0.1 ps^{-1}. Pulling simulations were performed at pulling speeds ranging between 2e-5 nm/ps and 2e-3 nm/ps, with a 10 $kJ/mol/nm^2$ spring constant for the harmonic potential applied on the distance between the C- and N-termini of the protein. Alternatively, the same procedure was followed to pull between the N-terminus and the center of mass of a selected group of residues around helices III and IV whose mutation result in a strong decrease of the affinity for actin according to Hatschel et al ([19]; specifically, residues R1078, K1080, F1081, K1089, D1113 and K1116, using the Uniprot KB sequence indexing). For each pulling speed we run up to 50 simulation trajectories.

Simulations were analyzed in order to retrieve the maximum pulling force that results in an unfolding event for the protein. In order to do this, we estimated the fraction of native contacts (*Q*) and identified force peaks after filtering in the transition path region between high Q (folded) and low Q (unfolded) values.

Supplementary References

1. Khatri, A., Wang, J. & Pendergast, A.M. Multifunctional Abl kinases in health and disease. *J Cell Sci* **129**, 9-16 (2016).
2. Tse, J.R. & Engler, A.J. Preparation of hydrogel substrates with tunable mechanical properties. *Current protocols in cell biology / editorial board, Juan S. Bonifacino ... [et al.]* **Chapter 10**, Unit 10 16 (2010).
3. Echarri, A. *et al.* Caveolar domain organization and trafficking is regulated by Abl kinases and mDia1. *J Cell Sci* **125**, 3097-3113 (2012).
4. Hotulainen, P. & Lappalainen, P. Stress fibers are generated by two distinct actin assembly mechanisms in motile cells. *J Cell Biol* **173**, 383-394 (2006).
5. Oakes, P.W., Beckham, Y., Stricker, J. & Gardel, M.L. Tension is required but not sufficient for focal adhesion maturation without a stress fiber template. *J Cell Biol* **196**, 363-374 (2012).
6. Itoh, T. *et al.* Dynamin and the actin cytoskeleton cooperatively regulate plasma membrane invagination by BAR and F-BAR proteins. *Dev Cell* **9**, 791-804 (2005).
7. Ariotti, N. *et al.* Modular Detection of GFP-Labeled Proteins for Rapid Screening by Electron Microscopy in Cells and Organisms. *Dev Cell* **35**, 513-525 (2015).
8. Li, F. & Higgs, H.N. The mouse Formin mDia1 is a potent actin nucleation factor regulated by autoinhibition. *Curr Biol* **13**, 1335-1340 (2003).
9. Edelstein, A.D. *et al.* Advanced methods of microscope control using muManager software. *Journal of biological methods* **1** (2014).
10. Tolic-Norrelykke, S.F., Rasmussen, M.B., Pavone, F.S., Berg-Sorensen, K. & Oddershede, L.B. Stepwise bending of DNA by a single TATA-box binding protein. *Biophys J* **90**, 3694-3703 (2006).
11. Popa, I., Kosuri, P., Alegre-Cebollada, J., Garcia-Manyes, S. & Fernandez, J.M. Force dependency of biochemical reactions measured by single-molecule force-clamp spectroscopy. *Nature protocols* **8**, 1261-1276 (2013).
12. Hutter, J.L. & Bechhoefer, J. Calibration of atomic-force microscope tips. *Review of Scientific Instruments* **64**, 1868-1873 (1993).
13. Bustamante, C., Marko, J.F., Siggia, E.D. & Smith, S. Entropic elasticity of lambda-phage DNA. *Science* **265**, 1599-1600 (1994).
14. Ainavarapu, S.R. *et al.* Contour length and refolding rate of a small protein controlled by engineered disulfide bonds. *Biophys J* **92**, 225-233 (2007).
15. Alegre-Cebollada, J., Badilla, C.L. & Fernandez, J.M. Isopeptide bonds block the mechanical extension of pili in pathogenic Streptococcus pyogenes. *J Biol Chem* **285**, 11235-11242 (2010).
16. Karanicolas, J. & Brooks, C.L., 3rd The origins of asymmetry in the folding transition states of protein L and protein G. *Protein Sci* **11**, 2351-2361 (2002).
17. Graham, T.G. & Best, R.B. Force-induced change in protein unfolding mechanism: discrete or continuous switch? *The journal of physical chemistry. B* **115**, 1546-1561 (2011).
18. Hess, B., Kutzner, C., van der Spoel, D. & Lindahl, E. GROMACS 4: Algorithms for Highly Efficient, Load-Balanced, and Scalable Molecular Simulation. *Journal of chemical theory and computation* **4**, 435-447 (2008).
19. Hantschel, O. *et al.* Structural basis for the cytoskeletal association of Bcr-Abl/c-Abl. *Mol Cell* **19**, 461-473 (2005).
