# Supplementary material for: An Abl-FBP17 mechanosensing system couples local plasma membrane curvature and stress fiber remodeling during mechanoadaptation
Source: Nat Commun. 2019 Dec 20;10:5828. doi: 10.1038/s41467-019-13782-2 (PMC6925243; doi:10.1038/s41467-019-13782-2)
Supplement: Supplementary file 4 — Supplementary Data 1 [file 41467_2019_13782_MOESM4_ESM.docx]

**SUPPLEMENTARY DATA 1.**

Amino acid sequence of the polyprotein (I91ΔCys)_2_-ABD-(I91ΔCys)_2_

(I91ΔCys)_2_-ABD-(I91ΔCys)_2_

MRGSHHHHHHGSLIEVEKPLYGVEVFVGETAHFEIELSEPDVHGQWKLKGQPLAASPDAEIIEDGKKHILILHNAQLGMTGEVSFQAANTKSAANLKVKELRSLIEVEKPLYGVEVFVGETAHFEIELSEPDVHGQWKLKGQPLAASPDAEIIEDGKKHILILHNAQLGMTGEVSFQAANTKSAANLKVKELRSSTRVSLRKTRQPPERIASGTITKGVVLDSTEALCLAISRNSEQMASHSAVLEAGKNLYTFCVSYVDSIQQMRNKFAFREAINKLESNLRELQICPATASSGPAATQDFSKLLSSVKEISDIVRRRSLIEVEKPLYGVEVFVGETAHFEIELSEPDVHGQWKLKGQPLAASPDAEIIEDGKKHILILHNAQLGMTGEVSFQAANTKSAANLKVKELRSLIEVEKPLYGVEVFVGETAHFEIELSEPDVHGQWKLKGQPLAASPDAEIIEDGKKHILILHNAQLGMTGEVSFQAANTKSAANLKVKELRSCC

Equivalent sequence in 1zzp

STRVSLRKTRQPPERIA is not found in pdb and is considered to be unfolded in the calculations of unfolding length.
