# Supplementary material for: An Abl-FBP17 mechanosensing system couples local plasma membrane curvature and stress fiber remodeling during mechanoadaptation
Source: Nat Commun. 2019 Dec 20;10:5828. doi: 10.1038/s41467-019-13782-2 (PMC6925243; doi:10.1038/s41467-019-13782-2)
Supplement: Supplementary file 5 — Reporting Summary [file 41467_2019_13782_MOESM5_ESM.pdf]

nature research

Corresponding author(s): DBPR NCOMMS-19-30999-A

Last updated by author(s): Nov 13, 2019

Reporting Summary

Nature Research wishes to improve the reproducibility of the work that we publish. This form provides structure for consistency and transparency in reporting. For further information on Nature Research policies, see Authors & Referees and the Editorial Policy Checklist.

Statistics

For all statistical analyses, confirm that the following items are present in the figure legend, table legend, main text, or Methods section.

n/a	Confirmed	
☐	☒	The exact sample size (*n*) for each experimental group/condition, given as a discrete number and unit of measurement
☐	☐	A statement on whether measurements were taken from distinct samples or whether the same sample was measured repeatedly
☐	☒	The statistical test(s) used AND whether they are one- or two-sided<br>*Only common tests should be described solely by name; describe more complex techniques in the Methods section.*
☐	☐	A description of all covariates tested
☐	☐	A description of any assumptions or corrections, such as tests of normality and adjustment for multiple comparisons
☐	☒	A full description of the statistical parameters including central tendency (e.g. means) or other basic estimates (e.g. regression coefficient) AND variation (e.g. standard deviation) or associated estimates of uncertainty (e.g. confidence intervals)
☐	☐	For null hypothesis testing, the test statistic (e.g. *F*, *t*, *r*) with confidence intervals, effect sizes, degrees of freedom and *P* value noted<br>*Give P values as exact values whenever suitable.*
☐	☐	For Bayesian analysis, information on the choice of priors and Markov chain Monte Carlo settings
☐	☐	For hierarchical and complex designs, identification of the appropriate level for tests and full reporting of outcomes
☐	☐	Estimates of effect sizes (e.g. Cohen's *d*, Pearson's *r*), indicating how they were calculated

Our web collection on statistics for biologists contains articles on many of the points above.

Software and code

Policy information about availability of computer code

Data collection	Leica LAS AF software, Carl Zeiss ZEN, EM-MENU 4.0, Micro-manager, Igor 6.
Data analysis	Excel, Definiens, GraphPad Prism, Gromacs version 4.0.5, Python, Image J, Igor 6.

For manuscripts utilizing custom algorithms or software that are central to the research but not yet described in published literature, software must be made available to editors/reviewers. We strongly encourage code deposition in a community repository (e.g. GitHub). See the Nature Research guidelines for submitting code & software for further information.

Data

Policy information about availability of data

All manuscripts must include a data availability statement. This statement should provide the following information, where applicable:

- Accession codes, unique identifiers, or web links for publicly available datasets
- A list of figures that have associated raw data
- A description of any restrictions on data availability

Data supporting the findings of this manuscript are available from the corresponding author upon reasonable request.

Field-specific reporting

Please select the one below that is the best fit for your research. If you are not sure, read the appropriate sections before making your selection.

☒ Life sciences ☐ Behavioural & social sciences ☐ Ecological, evolutionary & environmental sciences

For a reference copy of the document with all sections, see nature.com/documents/nr-reporting-summary-flat.pdf

Life sciences study design

All studies must disclose on these points even when the disclosure is negative.

Sample size	The sample size was set by the clarity of the phenotype. The lower limit of the sample size was set so it was sufficient to observe clearly the differences (p value below 0.05), while the upper limit was set so it was easily reproducible at a reasonable time for an independent lab. In experiments were a tendency was observed with a small sample size, sample size was increased to determine whether the tendency was statistically significant or not. In some cases the tendency was confirmed and validated statistically while in other cases it was not, in which case the sample size was not further increased.
Data exclusions	In figure 7d, one data point was excluded from the analysis as it deviated >7 SD away from the mean. Exclusion criteria were not pre-established.
Replication	All attempts at replication were successful for the experiments present in the paper.
Randomization	There was no need to allocate samples to different groups in this study. The samples, in our case cells, are separated into groups to undergo different treatments. These separate groups originate from the same pool of cells and the separation is random, i.e., cells are divided into different plates that represent a mixture of the same original population, thus plates assigned to different treatments are equivalent and thereby there is no need for randomization.
Blinding	In most experiments, the analysis, but not the acquisition, was blinded. In some cases the analysis required identification of transfected cells using different GFP-fusion proteins with distinct activities, which were evident to the observer in each condition, thus it could not be performed in a blind manner.

Reporting for specific materials, systems and methods

We require information from authors about some types of materials, experimental systems and methods used in many studies. Here, indicate whether each material, system or method listed is relevant to your study. If you are not sure if a list item applies to your research, read the appropriate section before selecting a response.

Materials & experimental systems

n/a	Involved in the study	
☐	☒	Antibodies
☐	☒	Eukaryotic cell lines
☐	☐	Palaeontology
☐	☐	Animals and other organisms
☐	☐	Human research participants
☐	☐	Clinical data

Methods

n/a	Involved in the study	
☐	☐	ChIP-seq
☐	☐	Flow cytometry
☐	☐	MRI-based neuroimaging

Antibodies

Antibodies used	Rabbit-made antibodies: caveolin (Becton Dickinson, cat # 610060); Cav1 XP (Cell Signaling Technology, cat # 3267, clone D46G3, lot # 6); FBP17 (gift from Pietro De Camilli lab, DOI: 10.1016/j.devcel.2005.11.005); FBP17 790 (Bethyl Laboratories, cat # A302-790A, lot # A302-790A-1); Phospho-CrkII Y221 (Cell Signaling Technology, cat # 3491, lot # 2); pacsin2 (kindly provided by Richard Lundmark); SNX9 (Sigma, cat # HPA031410, lot # R31792) and c-Abl (Santa Cruz, cat # sc-131, clone K12, lot # D2215). Mouse monoclonal antibodies: CrkII (Becton Dickinson, cat # 610035, clone 22/crk, lot # 5233714); CIP4 (Becton Dickinson, cat # 612556, lot # 23687); c-Abl (Becton Dickinson, clone 8E9, cat # 554148, lot # 52136); anti-phosphotyrosine (Millipore, clone 4G10, cat # 05-321, lot # 3156756); α-tubulin (Sigma, clone DM1A, cat # T-9026; GFP (Roche, clone 7.1+13.1, cat # 11814460, lot # 36405300); Myc (Santa Cruz Biotechnology, clone 9E10, cat # sc-40, lot # G050); Toca1 (a gift from Giorgio Scita, DOI: 10.1016/j.devcel.2014.08.006).
Validation	Anti-Caveolin: This antibody has been extensively used in the caveolae field (DOI: 10.1242/jcs.090134).<br>Anti-Cav1 XP: Specificity is provided by a western blot using Cav1 KO cell lysates and control lysates. The result shows a 21 kDa band, which corresponds to the size of Cav1, only in the controls, as shown in the manufacturer´s website. An example of a publication using this antibody in wild type and Cav1 KO cells is provided: DOI: 10.1016/j.celrep.2018.10.024.<br>Anti-FBP17: An immunoreactive band of the correct size is reduced upon specific silencing of FBP17 with two independent siRNAs (shown in figure S1a). Similarly, FBP17 gene edited cells (FBP17 KO cells) do not show immunoreactive bands of similar size to FBP17 (Fig. S1e –in Hela cells- and Fig. 3d -in RPE-1 cells-). Similarly, in FBP17 KO cells the staining of FBP17 is significantly reduced, compared to wild type cells (Fig. S1b-d). This antibody has been characterized also in DOI: 10.1016/j.devcel.2005.11.005.<br>Anti-FBP17 790: Western blot and immunoprecipitation assays using human cells are shown in the manufacturer´s website. In both cases a single band is observed corresponding to the theoretical size of FBP17. Used in DOI: 10.1091/mbc.E15-04-0232.<br>Anti-Phospho-CrkII Y221: It does not react against other tyrosine-phosphorylated proteins and detects endogenous levels of CrkII only when phosphorylated at tyrosine 221. The antibody cross-reacts with Tyr207-phosphorylated Crk, as indicated in the manufacturer´s website.<br>Anti-Pacsin2: Specificity is shown in figure S1a of this study. An immunoreactive band of the correct size is reduced, compared to

the control, upon specific silencing of pacsin2.
Anti-SNX9: Specificity is shown in figure S1a of this study. An immunoreactive band of the correct size is reduced, compared to the control, upon specific silencing of SNX9.
Anti-c-Abl (K12). This antibody has been extensively used in the field, an example is shown in a representative publication, DOI: 10.1101/gad.13.18.2400.
Anti-CrkII: Western blot of human cell lysates is shown in the manufacturer´s website. A single band corresponding to the theoretical molecular weight of CrkII is shown.
Anti-CIP4: Specificity is shown in figure S1a of this study. An immunoreactive band of the correct size is reduced, compared to the control, upon specific silencing of CIP4.
Anti-c-Abl (8E9): The specificity of the antibody is shown in our previous publication. In c-Abl/Arg double KO cells, no band corresponding to c-Abl is observed, while in wild type cells a band with the correct theoretical molecular weight is observed (DOI:10.1242/jcs.090134).
Anti-Phosphotyrosine: This antibody is widely used and validated for western blot, application used in this study, as indicated in the manufacturer´s website.
Anti-α-tubulin: This antibody is widely used and validated for western blot, application used in this study, as indicated in the manufacturer´s website
Anti-GFP: This antibody is widely used and validated for IP and western blot, applications used in this study, as indicated in the manufacturer´s website.
Anti-Myc: This antibody is widely used and validated for IF and western blot, applications used in this study, as indicated in the manufacturer´s website.
Anti-Toca1: Specificity is shown in figure S1a of this study. An immunoreactive band of the correct size is reduced, compared to the control, upon specific silencing of Toca1.

Eukaryotic cell lines

Policy information about cell lines

Cell line source(s)	HeLa, RPE1, 293/17 and U2OS cells were purchased from ATCC. Human foreskin fibroblast were obtained from a healthy donor.
Authentication	None of the cell lines were autheticated as they were purchased from ATCC.
Mycoplasma contamination	All cell lines were screened for mycoplasma presence (with Mycoalert PLUS mycoplasma detection kit, Lonza) and the results were negative.
Commonly misidentified lines (See ICLAC register)	None.

Palaeontology

Specimen provenance	*Provide provenance information for specimens and describe permits that were obtained for the work (including the name of the issuing authority, the date of issue, and any identifying information).*
Specimen deposition	*Indicate where the specimens have been deposited to permit free access by other researchers.*
Dating methods	*If new dates are provided, describe how they were obtained (e.g. collection, storage, sample pretreatment and measurement), where they were obtained (i.e. lab name), the calibration program and the protocol for quality assurance OR state that no new dates are provided.*

☐ Tick this box to confirm that the raw and calibrated dates are available in the paper or in Supplementary Information.

Animals and other organisms

Policy information about studies involving animals; ARRIVE guidelines recommended for reporting animal research

Laboratory animals	*For laboratory animals, report species, strain, sex and age OR state that the study did not involve laboratory animals.*
Wild animals	*Provide details on animals observed in or captured in the field; report species, sex and age where possible. Describe how animals were caught and transported and what happened to captive animals after the study (if killed, explain why and describe method; if released, say where and when) OR state that the study did not involve wild animals.*
Field-collected samples	*For laboratory work with field-collected samples, describe all relevant parameters such as housing, maintenance, temperature, photoperiod and end-of-experiment protocol OR state that the study did not involve samples collected from the field.*
Ethics oversight	*Identify the organization(s) that approved or provided guidance on the study protocol, OR state that no ethical approval or guidance was required and explain why not.*

Note that full information on the approval of the study protocol must also be provided in the manuscript.

Human research participants

Policy information about studies involving human research participants

Population characteristics	*Describe the covariate-relevant population characteristics of the human research participants (e.g. age, gender, genotypic information, past and current diagnosis and treatment categories). If you filled out the behavioural & social sciences study design questions and have nothing to add here, write "See above."*
Recruitment	*Describe how participants were recruited. Outline any potential self-selection bias or other biases that may be present and how these are likely to impact results.*
Ethics oversight	*Identify the organization(s) that approved the study protocol.*

Note that full information on the approval of the study protocol must also be provided in the manuscript.

Clinical data

Policy information about clinical studies

All manuscripts should comply with the ICMJE guidelines for publication of clinical research and a completed CONSORT checklist must be included with all submissions.

Clinical trial registration	*Provide the trial registration number from ClinicalTrials.gov or an equivalent agency.*
Study protocol	*Note where the full trial protocol can be accessed OR if not available, explain why.*
Data collection	*Describe the settings and locales of data collection, noting the time periods of recruitment and data collection.*
Outcomes	*Describe how you pre-defined primary and secondary outcome measures and how you assessed these measures.*

ChIP-seq

Data deposition

- [ ] Confirm that both raw and final processed data have been deposited in a public database such as GEO.
- [ ] Confirm that you have deposited or provided access to graph files (e.g. BED files) for the called peaks.

Data access links<br>*May remain private before publication.*	*For "Initial submission" or "Revised version" documents, provide reviewer access links. For your "Final submission" document, provide a link to the deposited data.*
Files in database submission	*Provide a list of all files available in the database submission.*
Genome browser session<br>(e.g. UCSC)	*Provide a link to an anonymized genome browser session for "Initial submission" and "Revised version" documents only, to enable peer review. Write "no longer applicable" for "Final submission" documents.*

Methodology

Replicates	*Describe the experimental replicates, specifying number, type and replicate agreement.*
Sequencing depth	*Describe the sequencing depth for each experiment, providing the total number of reads, uniquely mapped reads, length of reads and whether they were paired- or single-end.*
Antibodies	*Describe the antibodies used for the ChIP-seq experiments; as applicable, provide supplier name, catalog number, clone name, and lot number.*
Peak calling parameters	*Specify the command line program and parameters used for read mapping and peak calling, including the ChIP, control and index files used.*
Data quality	*Describe the methods used to ensure data quality in full detail, including how many peaks are at FDR 5% and above 5-fold enrichment.*
Software	*Describe the software used to collect and analyze the ChIP-seq data. For custom code that has been deposited into a community repository, provide accession details.*

Flow Cytometry

Plots

Confirm that:

☐ The axis labels state the marker and fluorochrome used (e.g. CD4-FITC).

☐ The axis scales are clearly visible. Include numbers along axes only for bottom left plot of group (a 'group' is an analysis of identical markers).

☐ All plots are contour plots with outliers or pseudocolor plots.

☐ A numerical value for number of cells or percentage (with statistics) is provided.

Methodology

Sample preparation	*Describe the sample preparation, detailing the biological source of the cells and any tissue processing steps used.*
Instrument	*Identify the instrument used for data collection, specifying make and model number.*
Software	*Describe the software used to collect and analyze the flow cytometry data. For custom code that has been deposited into a community repository, provide accession details.*
Cell population abundance	*Describe the abundance of the relevant cell populations within post-sort fractions, providing details on the purity of the samples and how it was determined.*
Gating strategy	*Describe the gating strategy used for all relevant experiments, specifying the preliminary FSC/SSC gates of the starting cell population, indicating where boundaries between "positive" and "negative" staining cell populations are defined.*

☐ Tick this box to confirm that a figure exemplifying the gating strategy is provided in the Supplementary Information.

Magnetic resonance imaging

Experimental design

Design type	*Indicate task or resting state; event-related or block design.*
Design specifications	*Specify the number of blocks, trials or experimental units per session and/or subject, and specify the length of each trial or block (if trials are blocked) and interval between trials.*
Behavioral performance measures	*State number and/or type of variables recorded (e.g. correct button press, response time) and what statistics were used to establish that the subjects were performing the task as expected (e.g. mean, range, and/or standard deviation across subjects).*

Acquisition

Imaging type(s)	*Specify: functional, structural, diffusion, perfusion.*
Field strength	*Specify in Tesla*
Sequence & imaging parameters	*Specify the pulse sequence type (gradient echo, spin echo, etc.), imaging type (EPI, spiral, etc.), field of view, matrix size, slice thickness, orientation and TE/TR/flip angle.*
Area of acquisition	*State whether a whole brain scan was used OR define the area of acquisition, describing how the region was determined.*

Diffusion MRI ☐ Used ☐ Not used

Preprocessing

Preprocessing software	*Provide detail on software version and revision number and on specific parameters (model/functions, brain extraction, segmentation, smoothing kernel size, etc.).*
Normalization	*If data were normalized/standardized, describe the approach(es): specify linear or non-linear and define image types used for transformation OR indicate that data were not normalized and explain rationale for lack of normalization.*
Normalization template	*Describe the template used for normalization/transformation, specifying subject space or group standardized space (e.g. original Talairach, MNI305, ICBM152) OR indicate that the data were not normalized.*
Noise and artifact removal	*Describe your procedure(s) for artifact and structured noise removal, specifying motion parameters, tissue signals and physiological signals (heart rate, respiration).*
Volume censoring	*Define your software and/or method and criteria for volume censoring, and state the extent of such censoring.*

Statistical modeling & inference

Model type and settings	*Specify type (mass univariate, multivariate, RSA, predictive, etc.) and describe essential details of the model at the first and second levels (e.g. fixed, random or mixed effects; drift or auto-correlation).*
Effect(s) tested	*Define precise effect in terms of the task or stimulus conditions instead of psychological concepts and indicate whether ANOVA or factorial designs were used.*

Specify type of analysis: ☐ Whole brain ☐ ROI-based ☐ Both

Statistic type for inference<br>(See Eklund et al. 2016)	*Specify voxel-wise or cluster-wise and report all relevant parameters for cluster-wise methods.*
Correction	*Describe the type of correction and how it is obtained for multiple comparisons (e.g. FWE, FDR, permutation or Monte Carlo).*

Models & analysis

n/a	Involved in the study
☐	☐ Functional and/or effective connectivity
☐	☐ Graph analysis
☐	☐ Multivariate modeling or predictive analysis

Functional and/or effective connectivity	*Report the measures of dependence used and the model details (e.g. Pearson correlation, partial correlation, mutual information).*
Graph analysis	*Report the dependent variable and connectivity measure, specifying weighted graph or binarized graph, subject- or group-level, and the global and/or node summaries used (e.g. clustering coefficient, efficiency, etc.).*
Multivariate modeling and predictive analysis	*Specify independent variables, features extraction and dimension reduction, model, training and evaluation metrics.*
